# A World at Risk: Aggregating Development Trends to Forecast Global Habitat Conversion

**DOI:** 10.1371/journal.pone.0138334

**Published:** 2015-10-07

**Authors:** James R. Oakleaf, Christina M. Kennedy, Sharon Baruch-Mordo, Paul C. West, James S. Gerber, Larissa Jarvis, Joseph Kiesecker

**Affiliations:** 1 The Nature Conservancy, Development by Design Program, Fort Collins, Colorado, United States of America; 2 Institute on the Environment, University of Minnesota, St. Paul, Minnesota, United States of America; 3 Land Use and Global Environment (LUGE) Research Group, Liu Institute for Global Issues, University of British Columbia, Vancouver, British Columbia, Canada; University of New England, AUSTRALIA

## Abstract

A growing and more affluent human population is expected to increase the demand for resources and to accelerate habitat modification, but by how much and where remains unknown. Here we project and aggregate global spatial patterns of expected urban and agricultural expansion, conventional and unconventional oil and gas, coal, solar, wind, biofuels and mining development. Cumulatively, these threats place at risk 20% of the remaining global natural lands (19.68 million km^2^) and could result in half of the world’s biomes becoming >50% converted while doubling and tripling the extent of land converted in South America and Africa, respectively. Regionally, substantial shifts in land conversion could occur in Southern and Western South America, Central and Eastern Africa, and the Central Rocky Mountains of North America. With only 5% of the Earth’s at-risk natural lands under strict legal protection, estimating and proactively mitigating multi-sector development risk is critical for curtailing the further substantial loss of nature.

## Introduction

### World at Risk

**Fig 1:**
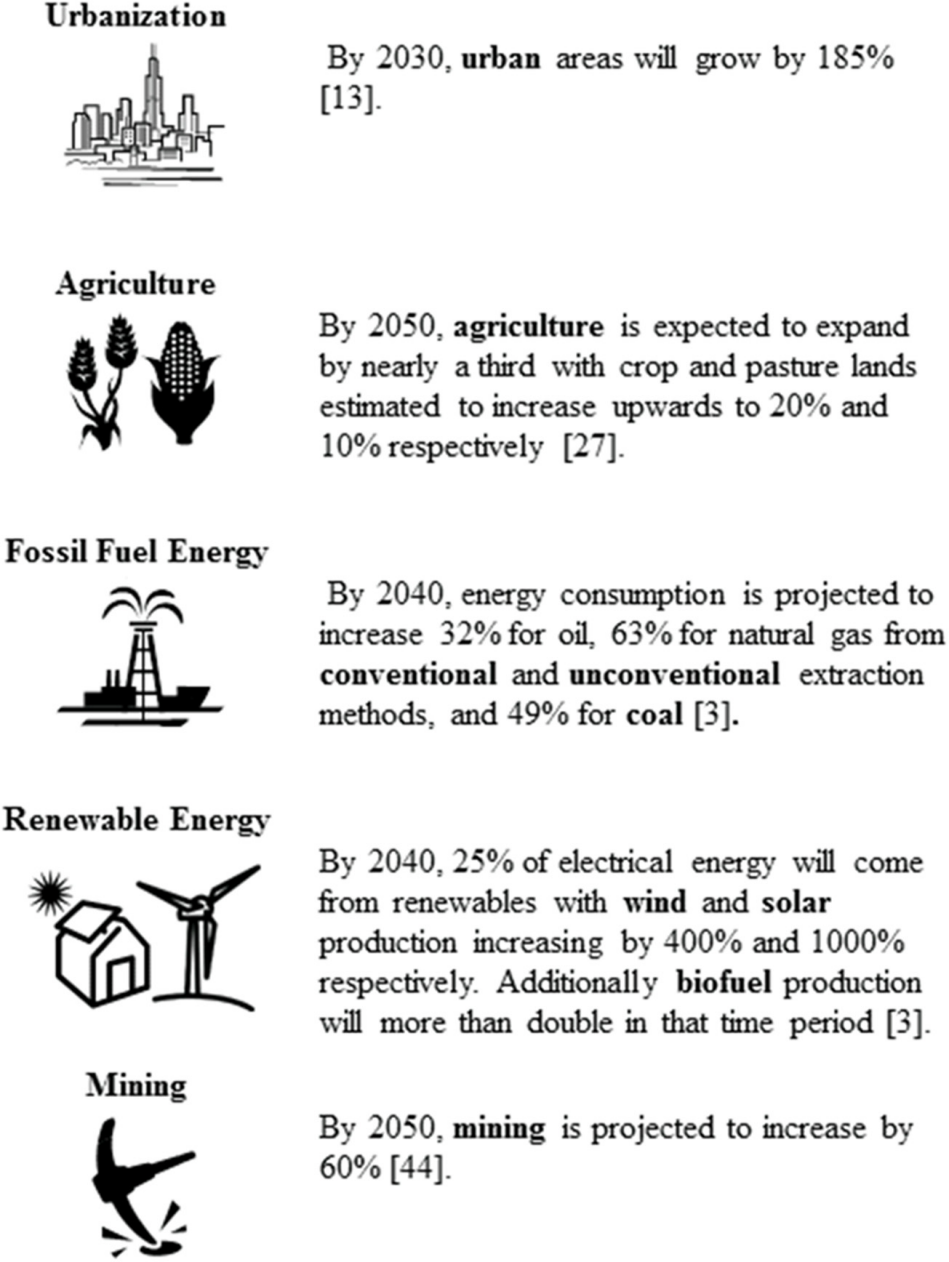
Global development pressures. Published estimates of potential expansion for the nine development sectors included into the cumulative development threat analysis (as indicated in bold).

Population increase, estimated to reach 9.6 billion by 2050 [1], along with gains in personal wealth and expansion of the middle class will continue to promote a rapid pace of development to meet the growing demands for food, water, housing, energy, minerals, and other resources [2,3] (Fig 1). For example, increasing demand for food and biofuels will result in nearly a billion new hectares of agricultural land by 2050 [4]. At the same time, this higher spending power of emerging markets is expected to increase global energy consumption by 56% in 2040 [3] and thus increase overall energy sprawl [5]. This pending development will help fuel economic growth, lift people out of poverty, and improve human living conditions [6,7], e.g. 1.7 billion more people are estimated to gain access to electricity by 2030 [8]. Given the expected benefits, development will likely go forward but by how much and where and at what cost to natural systems are unclear.

Proactively identifying habitats at risk of conversion and strategically balancing development objectives with conservation goals will be critical to achieve any semblance of sustainable development [9]. Previous studies have shed light on the current conditions of natural systems (e.g., refs [10–12]) while others have examined the global consequences of future habitat conversion from prominent sectors like agriculture and urbanization (e.g., refs [4,13–15]). We expand upon this foundational work and combine the potential impacts from multiple sectors to more comprehensively forecast future global development risk. Assessing cumulative risk is vital, because lack of risk due to one source of development is no guarantee of lack of risk from other sectors of development and having an understanding of where and how potential stressors overlap helps in mitigating these risks. We focus on urbanization, agriculture, energy, and mining as the major sources of land conversion and project nine forms of development for these drivers. Future resource development potentials for each of these nine sectors were spatially mapped, ranked, and aggregated globally to determine cumulative threat. We then examined patterns of high development risk, defined as the quarter of the globe with the highest cumulative threat scores overlapping natural areas, and examined these at-risk areas within geopolitical regions [16] and terrestrial biomes and ecoregions [17] to highlight opportunities for proactive and strategic conservation interventions.

## Results and Discussion

### Future patterns of development risk

**Fig 2:**
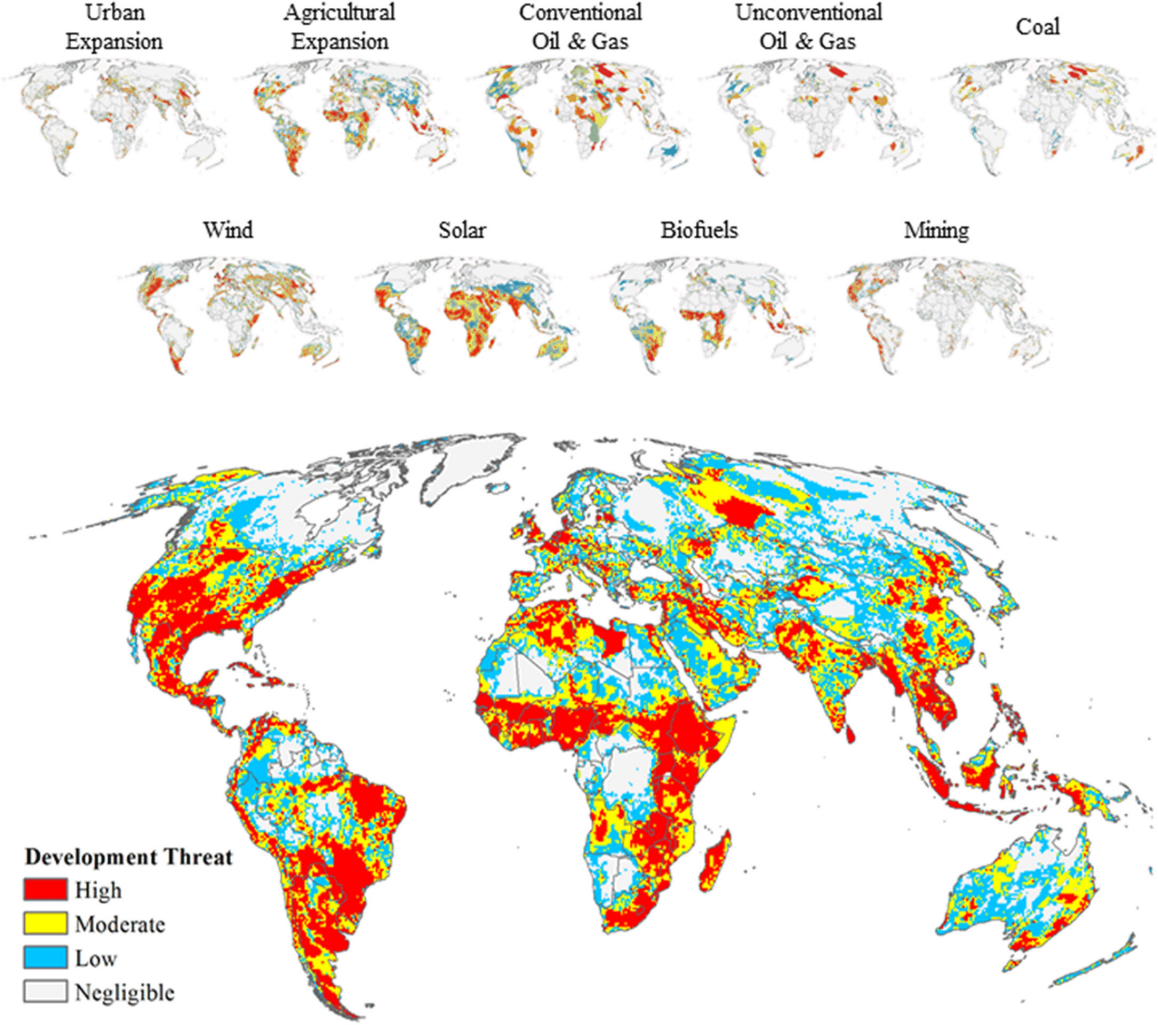
Future global development threat. Individual sector development threat maps (top and also shown in Figs 5–13) used to calculate the cumulative future development threat (bottom) identified by binning global lands (except Antarctica) into four equal-area categories with the “High” category defined as the quarter of the globe with the highest cumulative threat scores.

**Fig 3:**
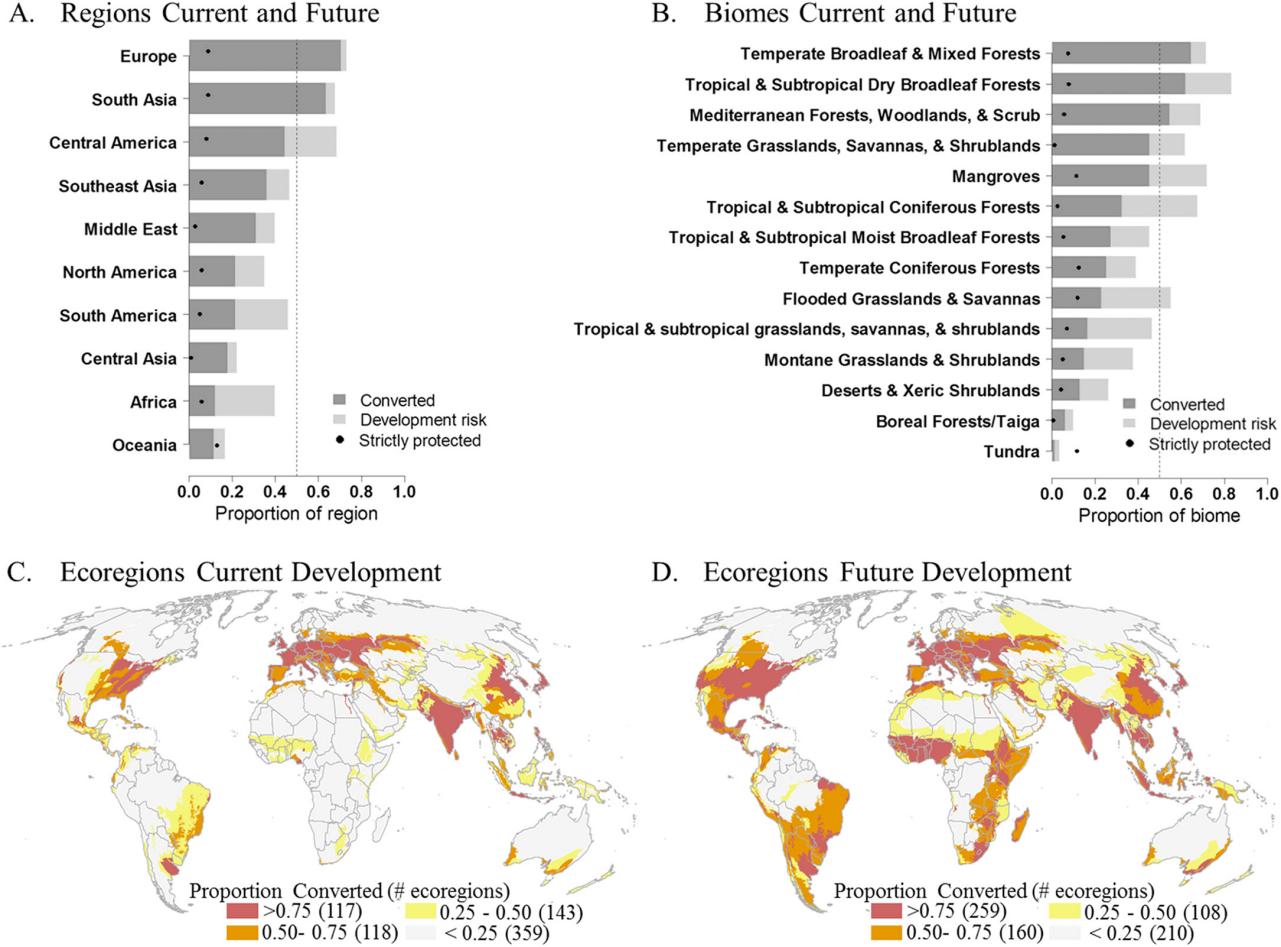
Proportion of land currently converted and future conversion per geopolitical region, biome, and ecoregion. The proportion of land in each geopolitical region (**A**) and biome (**B**) that is currently converted (dark grey), the proportion of natural lands at high risk to development (light grey), total future conversion (dark grey + light grey), and the proportion of strictly-protected natural lands at risk (dashed lines indicate the 50% threshold). Distribution of terrestrial ecoregions with > 0.75, 0.50, 0.25, and < 0.25 proportion of converted lands under (**C**) current conversion and (**D**) potential future land conversion including high development risk areas.

In the future, high threat to habitat conversion from the expansion of new development will be dispersed across the globe (Fig 2), which has the potential to impact 20% of the Earth’s remaining natural lands. The urgent need for managing future development is made evident by examining existing and future potential levels of habitat conversion. Our results suggest that the risk of conversion follows existing patterns of development with the three most converted regions, Central America, Europe and South Asia, remaining the most converted after accounting for future development risk (Fig 3A and S8 Table). In marked contrast, Africa and South America, which are currently among the least converted regions, also have the highest amount of land under potential development risk (8.18 and 4.32 million km^2^ for Africa and South America, respectively). Hence, when development risk is accounted for, the amount of converted lands could approximately double for South America and triple for Africa (S8 Table).

Currently, 21% of all biomes have half of their natural habitats converted and 57% have more than a quarter converted (Fig 3B). Future development could lead to half of the world’s biomes having more than 50% of their natural habitats converted, and all biomes (with the exception of Boreal Forests and Tundra) with over 25% of their natural lands at risk of conversion (Fig 3B). While development risk is highly dispersed globally, potential impacts are disproportionally borne by three biomes that contain 66% of delineated at-risk natural areas: Tropical and Subtropical Grasslands, Savannas, and Shrublands (5.98 million km^2^); Deserts and Xeric Shrublands (3.74 million km^2^); and Tropical and Subtropical Moist Broadleaf Forests (3.4 million km^2^) (S9 Table). Accounting for current and potential future development, three biomes could become predominantly human-modified: Tropical and Subtropical Dry Broadleaf Forests (83%), Mangroves (72%), and Temperate Broadleaf and Mixed Forests (71%)(Fig 3B and S9 Table).

**Fig 4:**
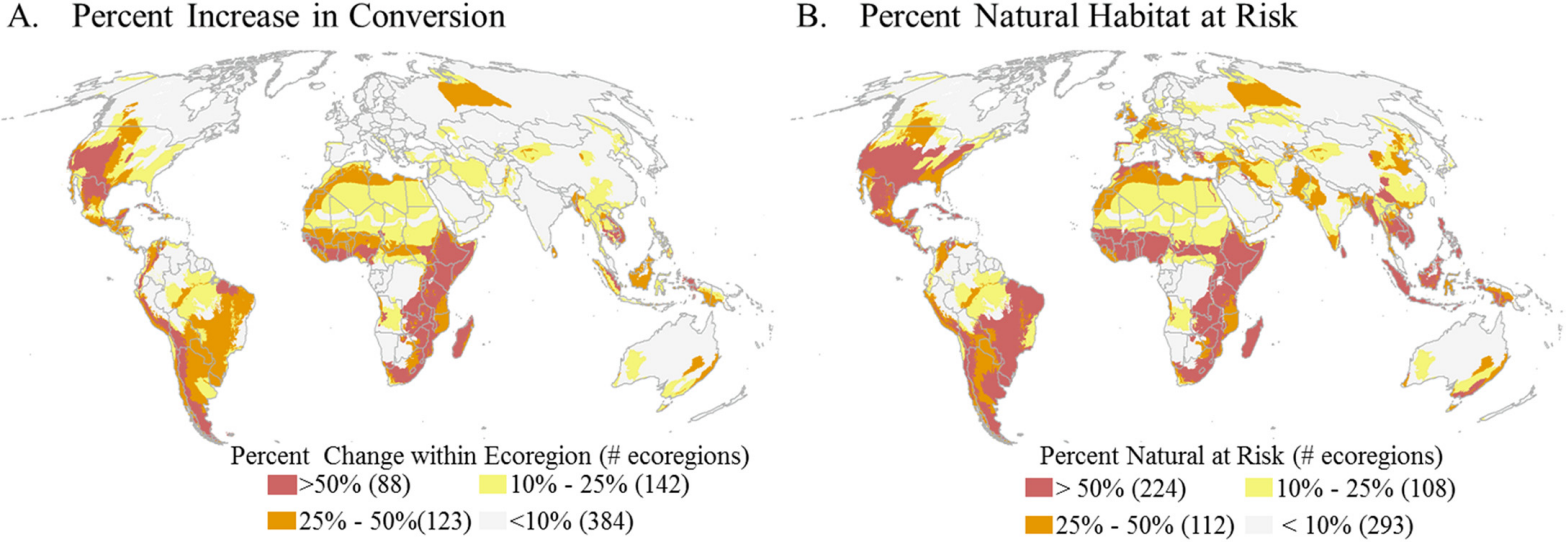
Ecoregions facing substantial change based on development risk to natural habitats. Distribution of ecoregions binned into four categories > 50, 25, 10, and < 10 percent displaying **A)** the potential percent change in conversion within an ecoregion from current to future and **B)** the percent natural habitat within an ecoregion at risk to future development.

When factoring high development risk at a finer scale, the number of ecoregions with 50% or more of land at risk of conversion nearly doubles from 235 ecoregions to 419 ecoregions with 142 additional ecoregions having the potential of 75% of the land being converted (Fig 2C and 2D, and S1 Dataset). Of these 142 ecoregions, 41 will shift from having less than 25% of the lands converted to over 75% (S1 Dataset) and overall 88 ecoregions will see a jump in conversion greater than 50% (Fig 4A). These substantial changes in conversion are projected for Central and Eastern Africa, Southern and Western South America and within the Central Rocky Mountain Region of North America (Figs 2C, 2D and 3A). When examining the potential conversion of what is currently natural, we identify 224 ecoregions that have 50% or more of natural habitat at risk to development (Fig 4B).

### Current land protection and mitigation policy are insufficient

**Fig 5:**
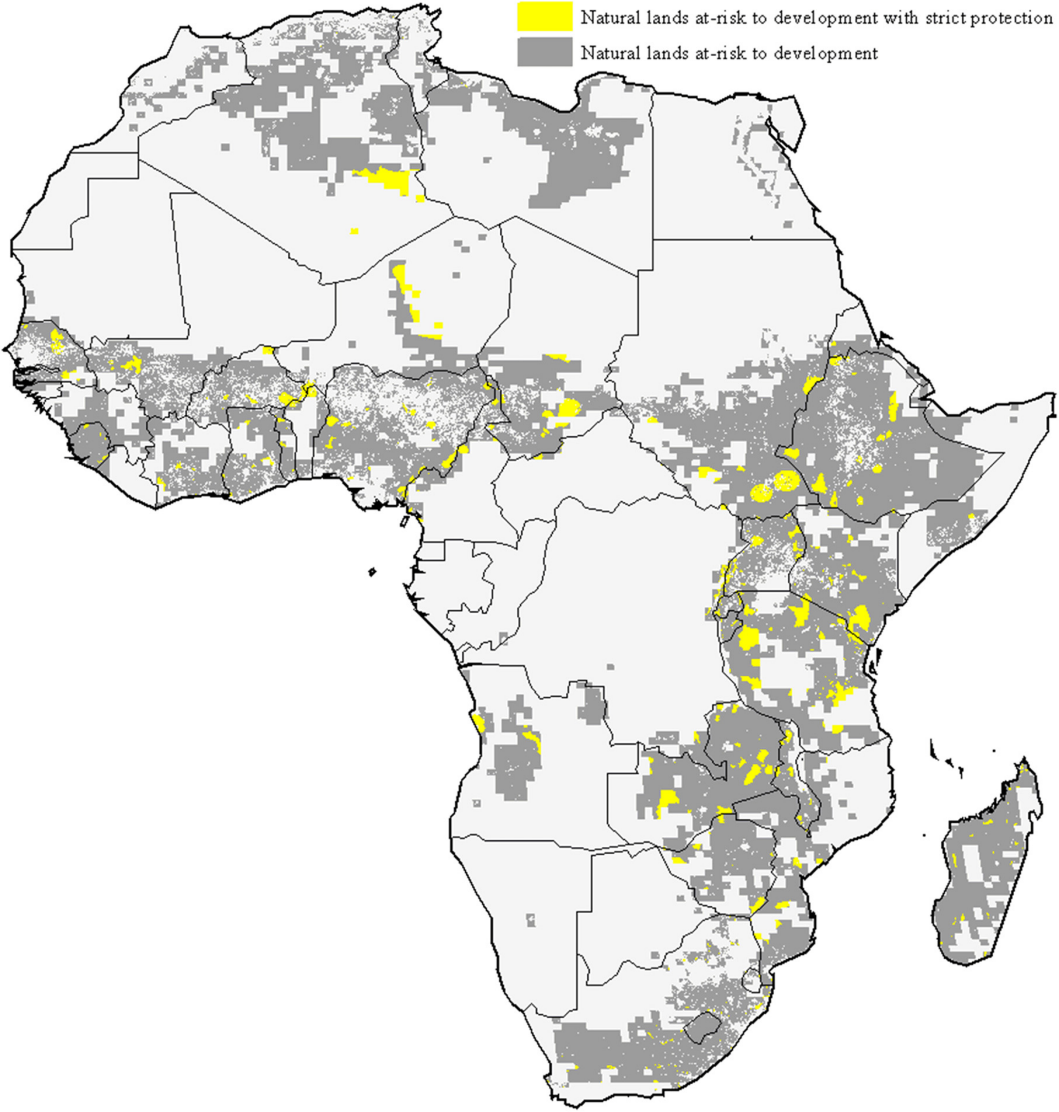
Africa natural lands at risk to future development. Africa natural lands at high risk to future development (grey and yellow) and current at-risk natural lands benefiting from strict legal protection (yellow only).

While habitat protection is an important conservation strategy [18], the current placement of protected areas is not well positioned to mitigate future development impacts. Globally, only 5% of the natural lands at high risk of development are under strict protection, defined as IUCN category 1–4 [19]. This lack of adequate protection for at-risk natural lands is apparent in biomes and regions subjected to high development risk (e.g. Fig 5).

Other policy tools available for countries to regulate development impacts are Environmental Impact Assessments (EIA) and impact mitigation. EIA in conjunction with impact mitigation are a systematic process that examines the environmental consequences of planned developments and emphasizes prediction and prevention of environmental damage through the application of the mitigation hierarchy: avoid, minimize, restore, or offset [20]. This process represents one of the best opportunities to incorporate environmental information into real world decision making [9,21]; however it is used predominately to regulate extractive industry activities, and in most countries does not address urbanization and agricultural expansion for food or biofuels. Additionally, mitigation tools are conventionally implemented through a narrow spatial lens: at a project- or site-level that often results in uncoordinated, piece-meal mitigation that fails to deliver conservation outcomes at relevant ecological scales [22]. This will make it challenging for EIA and mitigation strategies to address future cumulative development threats to natural systems since our analysis indicates that no single sector drives overall or region-specific development risk (S5 and S6 Tables). With the exception of urban expansion, all sectors are top-ranking contributors to cumulative development threat scores (S7 Table).

### Planning for the future: proactive mitigation

With development increasingly encroaching into more remote and previously undisturbed areas, it is critical that international corporations, governments and conservation organizations collaborate to reduce and minimize potential future impacts on remaining habitats. We propose that regulations for development siting and impact mitigation, as well as the implementation of land use planning, should target priority regions where development could threaten significant proportions of natural areas, such as the 224 ecoregions with the highest potential conversion of natural habitat (Fig 4B). These ecoregions could be further prioritized based on high biodiversity (e.g., refs [23–25]) and/or ecosystem service values (e.g., ref [26]). Once a priority region is identified, we suggest following analyses similar to ours that delineate natural areas at greatest risk to cumulative development threats, but to perform such analyses at finer (landscape) scales using more refined biodiversity data (e.g., as done in ref [27]). While our analysis provides an important global perspective, data uncertainties limit its use for most conservation interventions and mitigation planning efforts.

Implementation of mitigation requirements should also be conducted at landscape scales and include procedures for proactively evaluating the compatibility of proposed development with conservation goals to determine when impacts should be avoided and when development can proceed (e.g., as done in ref [21]). Given the expansive scale of expected impacts from a variety of sectors, developers will need to compensate for residual impacts through the use of biodiversity offsets. Also known as set-asides, compensatory habitat, or mitigation banks, biodiversity offsets are a tool for maintaining or enhancing environmental assets in situations where development is sought despite negative environmental impacts. To meet the need for additional investment in biodiversity offsets significant improvement of regulatory oversight will be needed [28].

Without strong oversight and proactive planning, countries containing high risk areas which also have weak governance and low levels of environmental protection are likely to suffer severe environmental damage [29]. In contrast, where environmental regulations are adequately enforced, impacts on biodiversity can be avoided and properly offset [21,27]. Opportunities for improvement include expanding, strategically locating, and enforcing global networks of protected areas in high-risk areas [18,30]; extending mitigation regulations to countries that currently lack them; and strengthening compliance where implementation of mitigation is weak [28]. In the interim, poorly performing national policies can be supplemented by the reinforcement of the mitigation hierarchy and adhering to planning mandates by multilateral development banks. For example, more than 70 Equator Principle financial institutions currently base their requirements on the International Finance Corporation’s (IFC) Performance Standards, which require that the projects they finance adhere to the mitigation hierarchy with regard to biodiversity and ecosystem service impacts [31]. In Africa and South America where development risk is high, the African and Inter-American Development Banks can provide leverage to ensure development projects avoid critical habitats and minimize and reduce impacts to less-critical areas and compensate where necessary.

While global agriculture, energy and mineral development are inevitable in the coming decades, their negative environmental impacts can be better managed. We suggest that using tools that cumulatively consider all current and future development threats, even when there are uncertainties and inaccuracies, will facilitate and advocate for more strategic and proactive development planning. This will allow for the world to better benefit from economic growth while also maintaining functioning ecosystems and critical biodiversity. It will however be critical to act proactively before development plans are cemented, and it becomes too late for these regions and biomes at greatest risk.

### Assumptions, limitations and uncertainties

Although our analysis identified natural lands at risk to development, we do not claim that all high risk lands will be converted nor espouse that these data should be used in site-level decision making. By summarizing at-risk lands at coarse scales, these data provide a basis by which to prioritize regions in need of conservation attention and to identify landscapes where finer-scale assessments should be conducted. We also caution that given threat area-ranking is relative; threat scores do not infer intensity of development (i.e. footprint) but rather indicates the relative likelihood that an area is expected to experience development and potential habitat conversion relative to the rest of the globe. Additionally, we assumed the higher likelihood of development from multiple threats in a region, the greater pressure for natural lands to be converted. This assumption does not take into consideration varying levels of impacts from each sector but rather treats each one equally. A lack of generalized impact measurements and the variation of our data sources forced this equal-weighting method.

We recognize that datasets used in our analysis often vary in terms of detail and resolution and that more detailed assessments may inflate development risk towards areas where these more detailed assessments exist (e.g. mineral resources). However investments in more detailed assessments often indicates the presence of economically viable resources and is a good indicator of potential development [32] so we were comfortable with the potential bias this might present. Additionally, we were limited to using publically available global data and thus relied on the accuracy and validation methods of those producing these data. For example, the IUCN WDPA [33] recognizes that not all protected areas are included in the database and inaccuracies related to protection level may be present. Moreover due to this global-lens, all threat assessments were first-order estimates of potential development. For example, we made the conservative assumption of linear growth for agricultural expansion and calculated it for each grid cell. Although the expansion may not be linear, the relative influences of other variables (e.g. demand, governmental policies, commodity prices, and prices) vary by location and time. These same variables could also influence any of the other threat assessments. However, to be consistent across the global analysis, we only use metrics that can be quantified using existing global data sets. Our simple, transparent approach can be easily modified for local analyses where better data exist. We also acknowledge that our assessment does not account for all conditions that can either promote development or occur in response to new developments; for example roads often comprise a significant aspect of development footprints [34]. We were however limited to measuring relative development threat for impacts which had publicly available and spatially explicit corresponding global datasets (e.g. future road locations). Again it would be critical for those assessing both development threats and protection at a more local-level to obtain data directly from a more definitive source than many of the global data repositories we relied on for our analysis and to tailor their analysis specifically for the region being modeled.

## Methods

Our analysis had two major components: 1) compiling individual and cumulative development threats globally, and 2) locating and prioritizing where cumulative threats pose a risk to terrestrial natural habitats. We projected development threats for nine sectors on terrestrial lands: urban and agricultural expansion, fossil fuels (conventional oil and gas, unconventional oil and gas, and coal), renewable energy (solar, wind, and biofuels), and mining. Future resource development potentials for each sector were created from publicly available global datasets (see S1 Table) and relatively ranked based on either the amount of unexploited resources (i.e. for fossil fuels, renewables, and mining) or estimated future area expansion derived from past trends (i.e. for urban and agriculture).

### Calculating individual and cumulative development threat

Sector development threat rankings were based on the locations of unexploited or potential resources necessary to support development and/or estimates of land predicted to be modified (S1 Table). More specifically, these relative threat assessments were derived from synthesizing fifty global datasets: urban and agricultural expansion (n = 3; refs [13,35,36]), fossil fuels (n = 30; refs [37–66]), renewables (n = 12; refs [67–79]), and mining (n = 5; refs [80–84]) and then for each sector aggregating values to a 50 km^2^ grid cell. This cell resolution was selected due to the varying scales of source data (S1 Table) and the flexibility this resolution provided for aggregation. Future threats from resource development potentials for each sector were relatively ranked from 1 to 100 across the globe, excluding Antarctica and any 50-km analysis grid cells with greater than 50% overlap with marine environments. We then summed the individual sectors scores to produce a cumulative global threat map (see section below on combining individual sector threats and Fig 2). We projected all spatial data to a Mollweide projection to minimize area distortion except for between-feature distance calculations in which we used the Two-Point Equidistance projection. Unless otherwise specified, we used ArcGIS v.10.2 with the Spatial Analyst Extension [85] to perform all spatial data development, procedures and analyses.

#### Area-ranked threat scores

We relatively ranked threat scores using an equal-area rank method [86,87]:

$$C_{r}=\frac{C_{i}+0.5f_{i}}{N}*100$$ (1)

where *C*_*r*_ is the ranking of the target cell value, *C*_*i*_ is the count of all grid cells with values less than the target cell value, *f*_*i*_ is the number of cells with the target cell value, and *N* is the total number of cells in the study extent. To ensure ranking consistency (i.e. top ranked cells all equal to 100) across sectors, we rescaled all equal-area rankings from 1 to 100. The area-ranking approach created uniform distributions of scores per sector, such that equal bin ranges represented an equal area on the globe, therefore allowing for similar distributions and equal weighting across threat sectors. Prior to our selecting the area-ranking approach we tried several normalization approaches (e. g., log, log-log, square-root, cubic and min-max scaling) [88], but these transformations failed to create normally distributed values from the generally right-skewed individual sector threat scores and caused some to have more weighting than others.

#### Urban expansion

**Fig 6:**
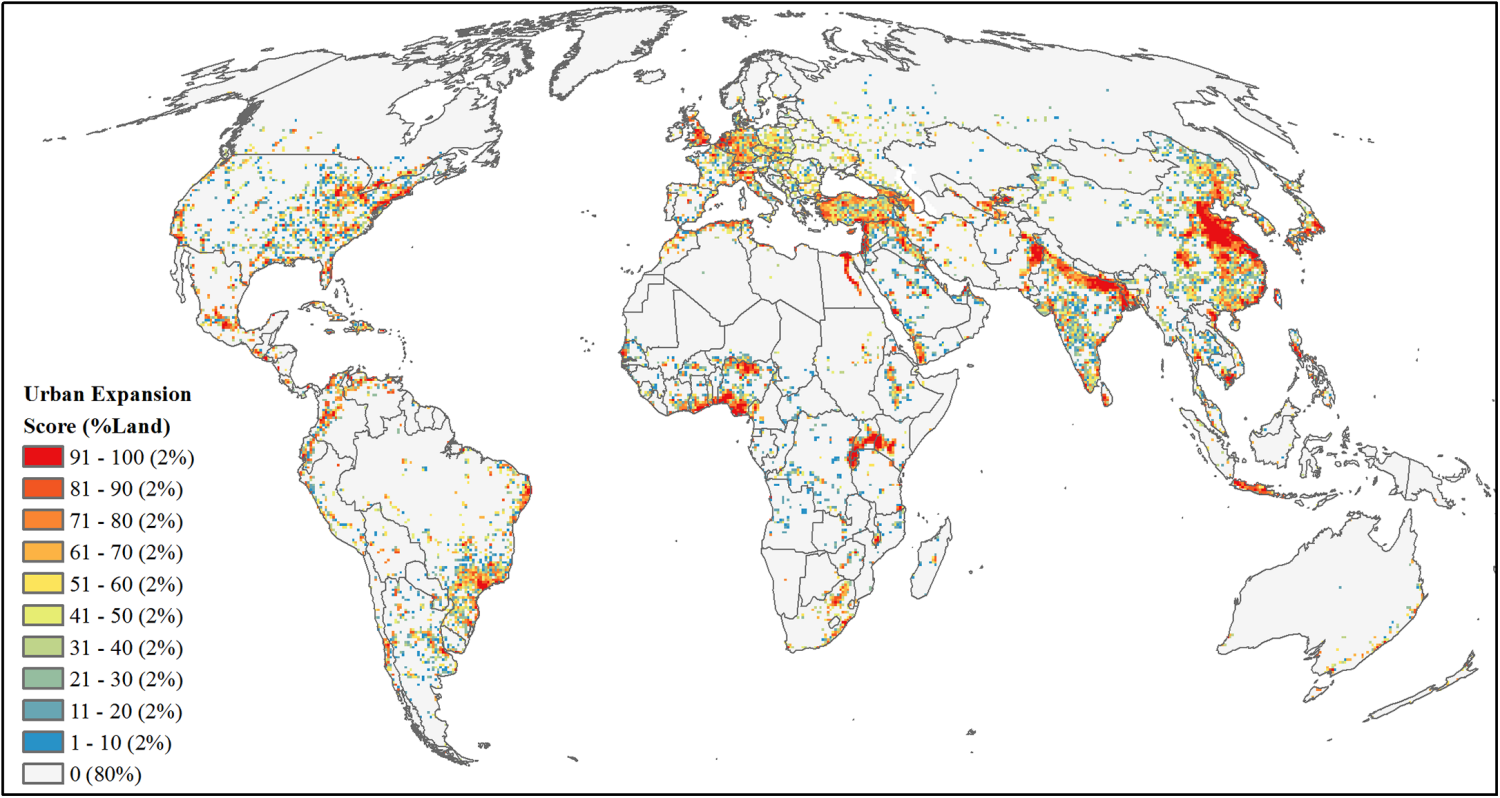
Projected future development threat of urban expansion. Area-ranked threat scores based on mean probabilities of global urban expansion by 2030, after excluding current urban areas.

We used published maps of urban expansion probabilities by 2030 [13] (S1 Table). Maps were developed by first forecasting an aggregate amount of urban expansion per defined global regions from probability density functions of projected GDP and urban population. Then the aggregate amount of expansion was spatially distributed using a spatially-explicit land-change model with covariates slope, distance to roads, population density, and land cover. Given that our analysis focused on future development expansion into existing natural areas, we excluded areas already classified as urban and calculated the mean urban expansion probabilities for each 50-km grid cell. We then area-ranked mean probabilities of urban expansion for an urban development threat score (Fig 6).

#### Agriculture expansion

**Fig 7:**
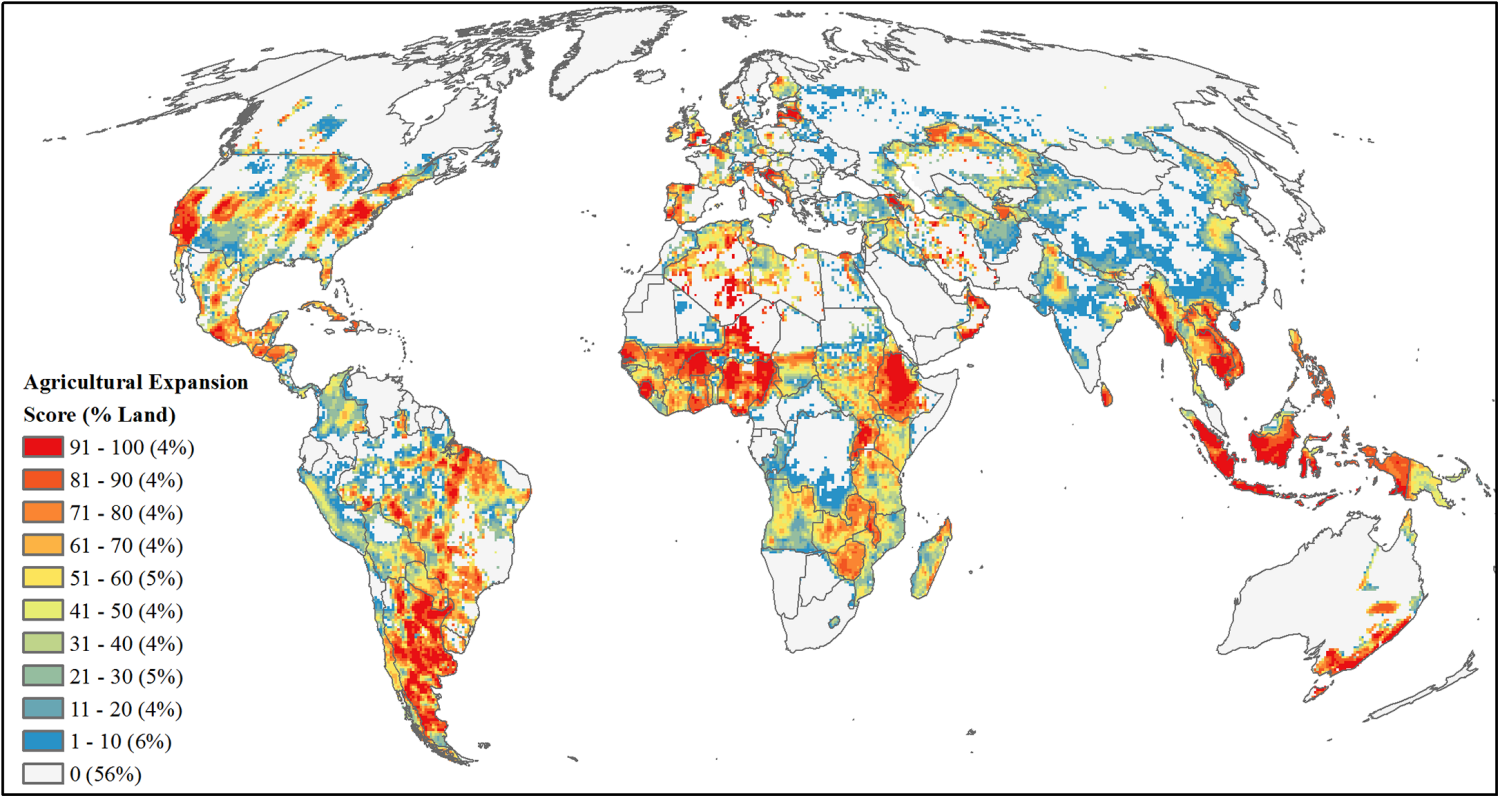
Projected future development threat of agricultural expansion. Area-ranked threat scores based on estimates of fractional amount of agricultural expansion by 2030 extrapolated from 2000–2011 cropland and pasture time series maps.

We calculated agriculture expansion rates using a 2000–2011 time series of global cropland and pasture maps following the methods of Ramankutty et al. [35] (S1 Table). We then: 1) summed for each year and 5 arc-minute (approx. 10x10-km) grid cell the total agricultural area in cropland and pasture (hereafter ag), 2) calculated the yearly fraction of area in ag within each grid cell, and 3) linearly regressed the 12-year time series and used the slope parameter as cell-specific rate of ag expansion. To focus on areas of potential development, we limited our analyses to only those cells with positive rates (slopes), and then averaged the rates of expansion within a resampled 50-km rectangle (corresponding to our threat analysis scale), which in effect accounted for higher likelihood of expansion into neighboring cells. To estimate the fractional area of agriculture expansion by 2030 for each 10-km grid cell, we resampled averaged rates back to a 10-km resolution and multiplied the averaged expansion rates by 19 (representing 19 years from 2012–2030). In cases where the fractional area of ag expansion for 2030, current ag land (i.e. 2011), and urban areas [36] summed to be greater than one (i.e., greater than the entire grid cell), we adjusted the fractional area of ag expansion as the maximum potential land conversion in the cell by subtracting the fractional areas of current ag and urban areas from one. Finally, we calculated the mean fractional area at a resampled 50-km grid resolution and area-ranked this mean value (Fig 7).

#### Conventional oil and gas

**Fig 8:**
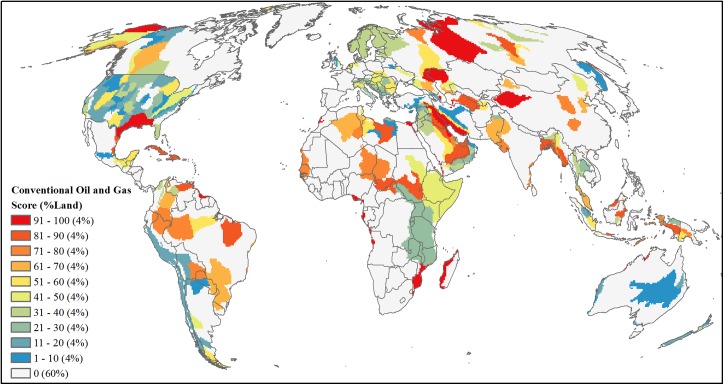
Projected future development threat of conventional oil and gas. Area-ranked threat scores based on province-level estimates of undiscovered million barrels of oil equivalent for oil, natural gas, and liquid natural gas resources.

For conventional oil and gas, our analysis used undiscovered volumes produced by the U.S. Geological Survey (USGS) for those global, geologic provinces which either currently contribute or are estimated to contribute in the future to the world’s reserves [37,39] (S1 Table). We augmented these global USGS assessments with more detailed national-level assessments available for the U.S. [38] and Australia [40], which resulted in a total of 305 geologic provinces worldwide with undiscovered oil and gas volume estimates. From this total, we excluded provinces with zero undiscovered volume (*n* = 8), and those provinces identified as having only offshore development [41,42] or not having at least 50% of the province overlapping land (*n* = 55). For each of the remaining 242 provinces we calculated the million barrels of oil equivalent (MBOE) of undiscovered oil, liquid natural gas, and natural gas with a conversion factor of 6 MBOE per each billion cubic feet of natural gas. We summed these values to quantify development potential per province and assigned this total MBOE value to overlapping 50-km grid cells with 50% or more of the cell intersecting a province. We then area-ranked cells based on this total MBOE value (Fig 8).

#### Unconventional oil and gas

**Fig 9:**
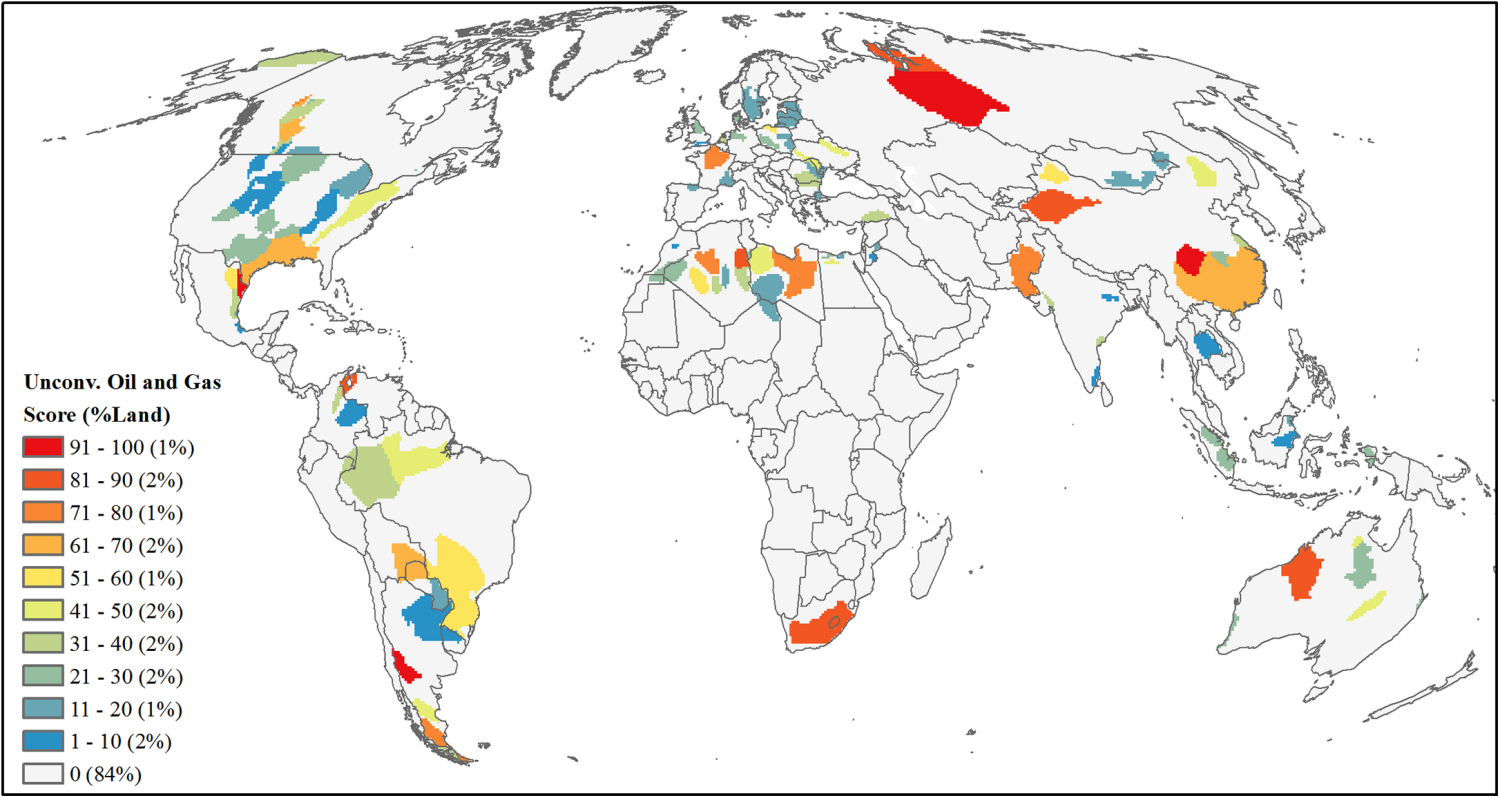
Projected future development threat of unconventional oil and gas. Area-ranked threat scores based on basin-level estimates of technically recoverable billion barrels of oil equivalent for unconventional oil, natural gas, and liquid natural gas resources.

For unconventional oil and gas, we focused on resources found in shale and other sedimentary formations but did not include any coal-bed methane resource as this latter form of development was estimated separately (see below). We used global [43] and U.S. [38] assessments of technically recoverable unconventional oil and natural gas (S1 Table). For non-U.S. regions, we geo-referenced and digitized basin maps from the global assessment [43] and linked resource volumes listed in the assessment to each basin (*n* = 98). For the U.S., we relied on spatially available data on basin location and resource estimate [38] (*n* = 17). We combined both datasets and for each basin, and converted all technically recoverable oil, natural gas and liquid natural gas volume estimates to billion barrels of oil equivalent (BBOEs) and summed these values for a total basin-specific resource estimate. Finally, we converted these basins to a raster with a 50-km resolution grid and area-ranked cells based on the summed BBOE value (Fig 9).

#### Coal

**Fig 10:**
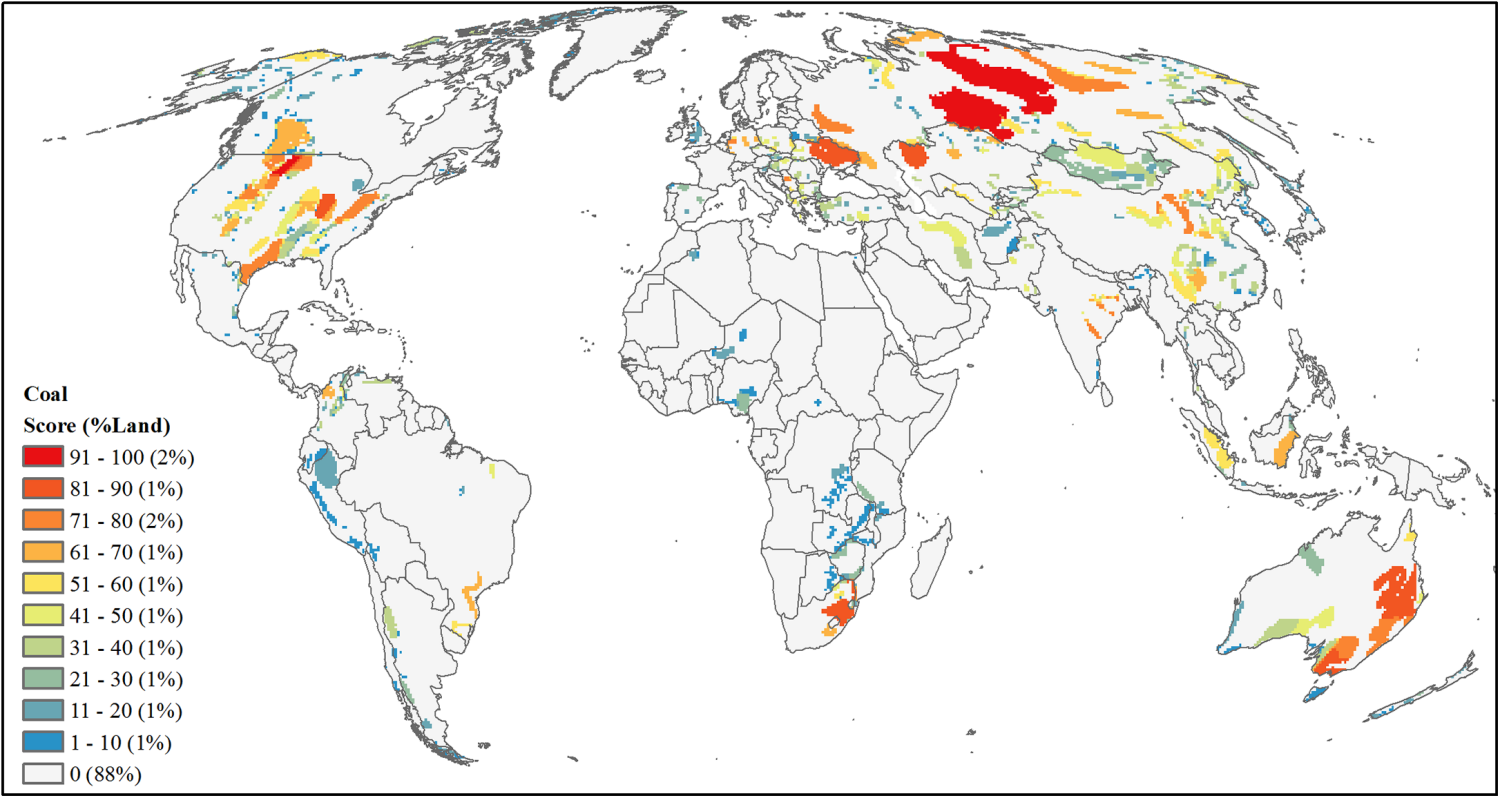
Projected future development threat of coal. Area-ranked threat scores based on coal basin reserve estimates in million short tons attributed form country- and state-level coal reserve data.

For coal, we combined tabular data of 2008 coal reserve estimates (million short tons) at the country-level [59] (S1 Table) with spatial data identifying coal-bearing areas for 65 countries (S2 Table). Spatially explicit data for 39 countries were available [44–48] while for the remaining 26 countries we geo-referenced existing digital maps and digitized coal-bearing areas [49,51–58,61–66] (S2 Table). We intersected all coal-bearing areas with the 65 country boundaries, calculated for each area its proportion that contributed to the countries’ overall total, and assigned individual coal reserve values per area by multiplying this proportion times the total country coal reserves. For four of the five top coal-producing countries (U.S., China, Australia, and India), we were able to further refine reserve estimates with published local government estimates [49,50,57,58]; we used the same attribution procedures based on proportion of overlap of coal-bearing areas with state or province boundaries and multiplied by the reserve estimates. Additionally 29 countries had coal-bearing areas but did not have any country or local reserve estimate. Due to these coal-bearing areas having some potential development threat, we assigned each of these coal-bearing areas with the lowest reserve value for all calculated areas of one thousand short-tons. Finally, we converted these coal-bearing areas to a raster with a 50-km resolution grid and area-ranked cells based on the reserve estimates (Fig 10).

#### Wind

We used three main characteristics to estimate wind power development: 1) wind resources, 2) land suitability based on accessibility and physical restriction, and 3) economic feasibility based on electricity demand and distribution. Following other utility-scaled wind siting analyses [89–91], we synthesized and scaled each characteristic separately on its likelihood to support wind development. For wind resources we used annual averaged wind speed measured as m/s at 80m above Earth’s surface [67] (S1 Table), and restricted the analyses to wind speeds ≥ 6.4 m/s identified as most feasible for utility-scaled development. We then min-max normalized these wind speeds from 0.01–1.

To create an overall binary land suitability map, we excluded land cover categories of rock and ice, artificial areas, water and wetlands [69], urban areas [36], and slopes > 20 degrees [70] and restricted all remaining lands to be within 80 km of an existing roads [71]. This produced an initial 300-m resolution raster identifying suitability that we resampled to 900-m resolution (3x3 cells) summing the binary results. Using a conservative approach where only resampled cells with a value of 9 (i.e. fully developed) were classified as suitable for wind power development, we resampled this result to 1 km through a simple bilinear process setting only those suitable cells to a value of 1.

To account for economic feasibility, we used proximity to demand centers and existing power plants based on the inverse Euclidean distances (i.e., smaller, straight-line distances result in higher feasibility) from large urban areas [72], defined as greater than 10,000 people, and current utility-scaled power producing locations [73] identified by power plants producing ≥ 5 MW (*n* = 15,782) and hydropower plants [74] (*n* = 1541). We created each distance raster using a Two-Point Equidistance projection, projected them to a Mollweide projection with a bilinear sampling technique, and then rescaled values from 0.001–1 with 1 being those cells nearest to the feature (i.e. large urban areas or power plants). These two distance raster datasets were combined by calculating the average for each cell. To account for possible government incentives and the proven ability to develop wind power, this average was then doubled for those cells falling within a country that already produces wind power [75,76]. For consistency with the other three factors, this final feasibility raster (with values ranging from 0.001 to 2) was then re-scaled back to 0.001 to 1.

**Fig 11:**
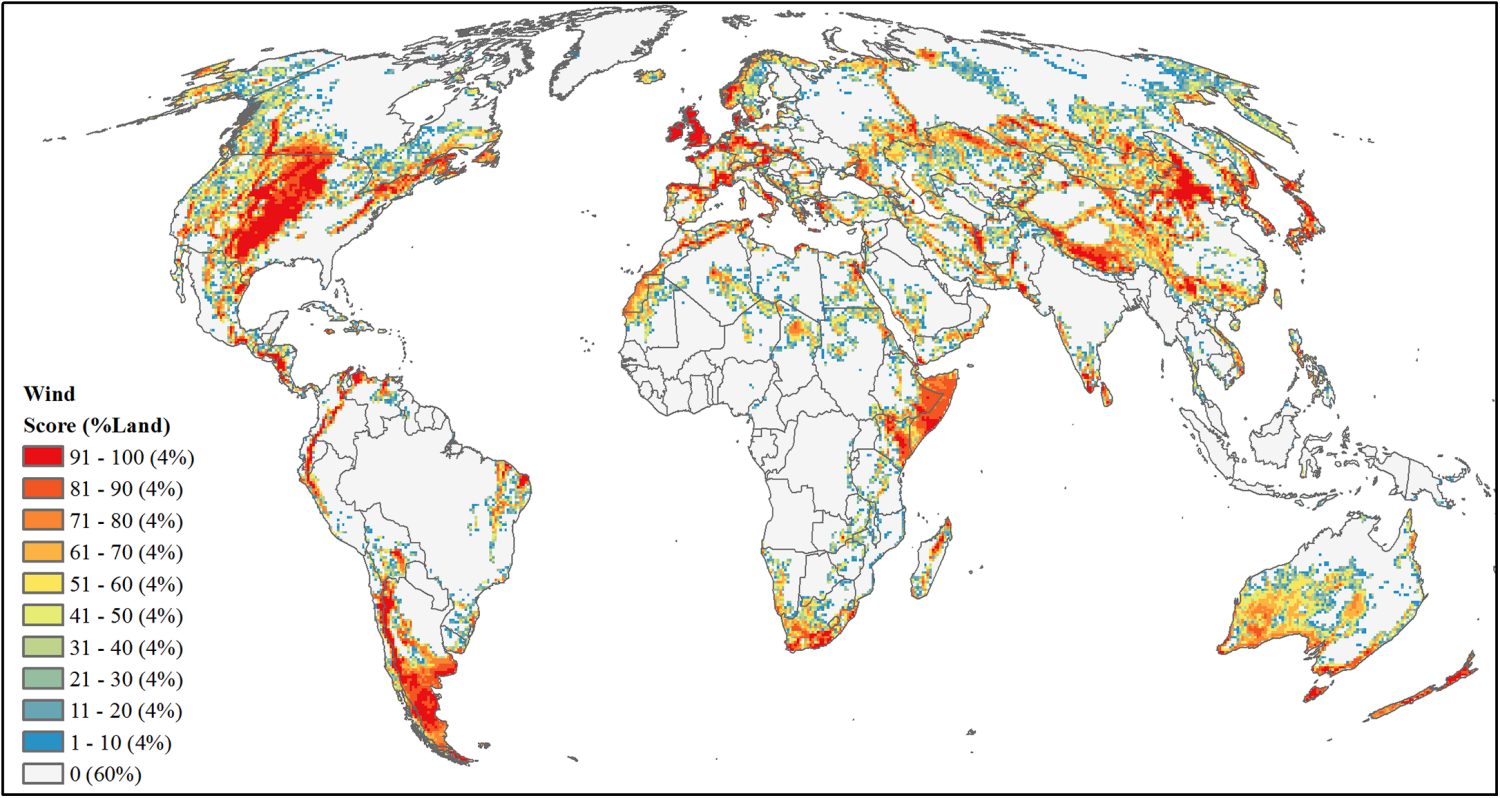
Projected future development threat of utility-scale wind power. Area-ranked threat scores based on combined metric of wind resources (m/s), land suitability, and economic feasibility for wind power development.

We combined the outputs layers of wind resources (5-km resolution), land suitability (1-km resolution), and economic feasibility (5-km resolution) by multiplying the three metrics into the final wind development threat score, maintaining a grid cell size of 1 km. We then resampled this product to a 50-km resolution grid summing all cell values from the smaller 1 km raster and area-ranked this summed value (Fig 11).

#### Solar

**Fig 12:**
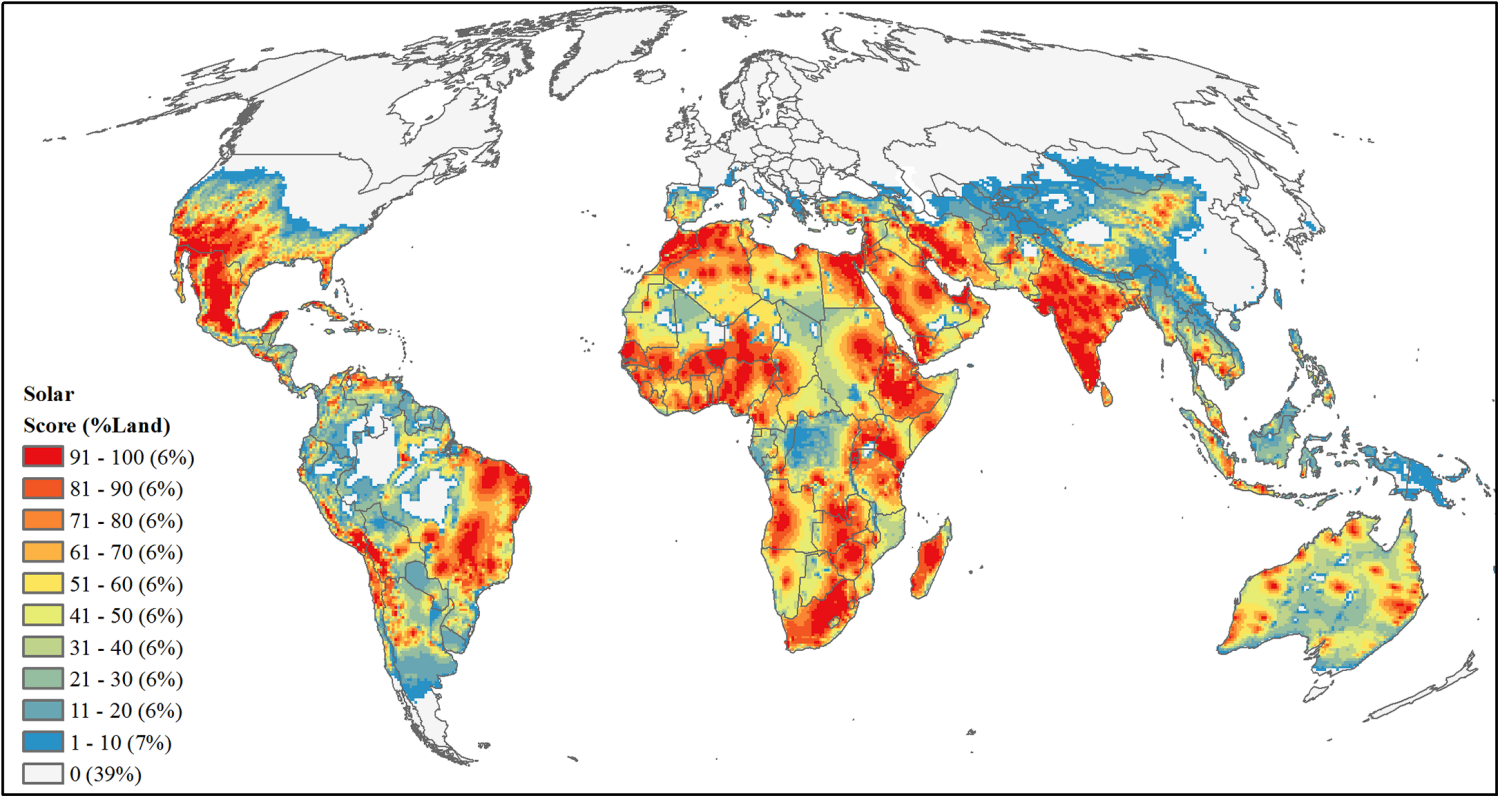
Projected future development threat of utility-scale solar power. Area-ranked threat scores based on combined metric of solar resources (W/m^2^), land suitability, and economic feasibility for solar power development.

For solar, we followed a similar approach to wind resources where we considered three main characteristics to estimate solar development: solar resources, land suitability, and economic feasibility. Utility-scaled solar power produces electricity using two main types of technologies: concentrating solar power (CSP) and photovoltaic (PV). Each technology is optimally implemented at different solar radiation levels measured as Global Horizontal Irradiance (GHI), where PV development is best implemented at GHI ≥ 182 Watts/m^2^ and CSP development is best implemented at GHI ≥ 217 W/m^2^. Therefore for solar resources, we used GHI data [68] (S1 Table) to create two solar resource grids, one for CSP and one for PV, where we included only cells with GHI values ≥ 217 and ≥182 W/m^2^, respectively. We then normalized values from 0.01–1 for each CSP and PV grid, summed the two resulting grids into one solar resource availability output, and normalized results again to a scale of 0.01–1. For the remaining procedures we followed the steps as described above for wind resources with two exceptions: slopes were classified as ≤ 3 degrees [91] and we doubled feasibility scores based on countries producing solar power [77,78]. Similarly to our wind threat, we multiplied the three development factors (i.e. solar resource, suitability and feasibility) to produce one solar development threat value, resampled this threat value to a 50-km resolution grid via summation, and area-ranked these summed values (Fig 12).

#### Biofuels

**Fig 13:**
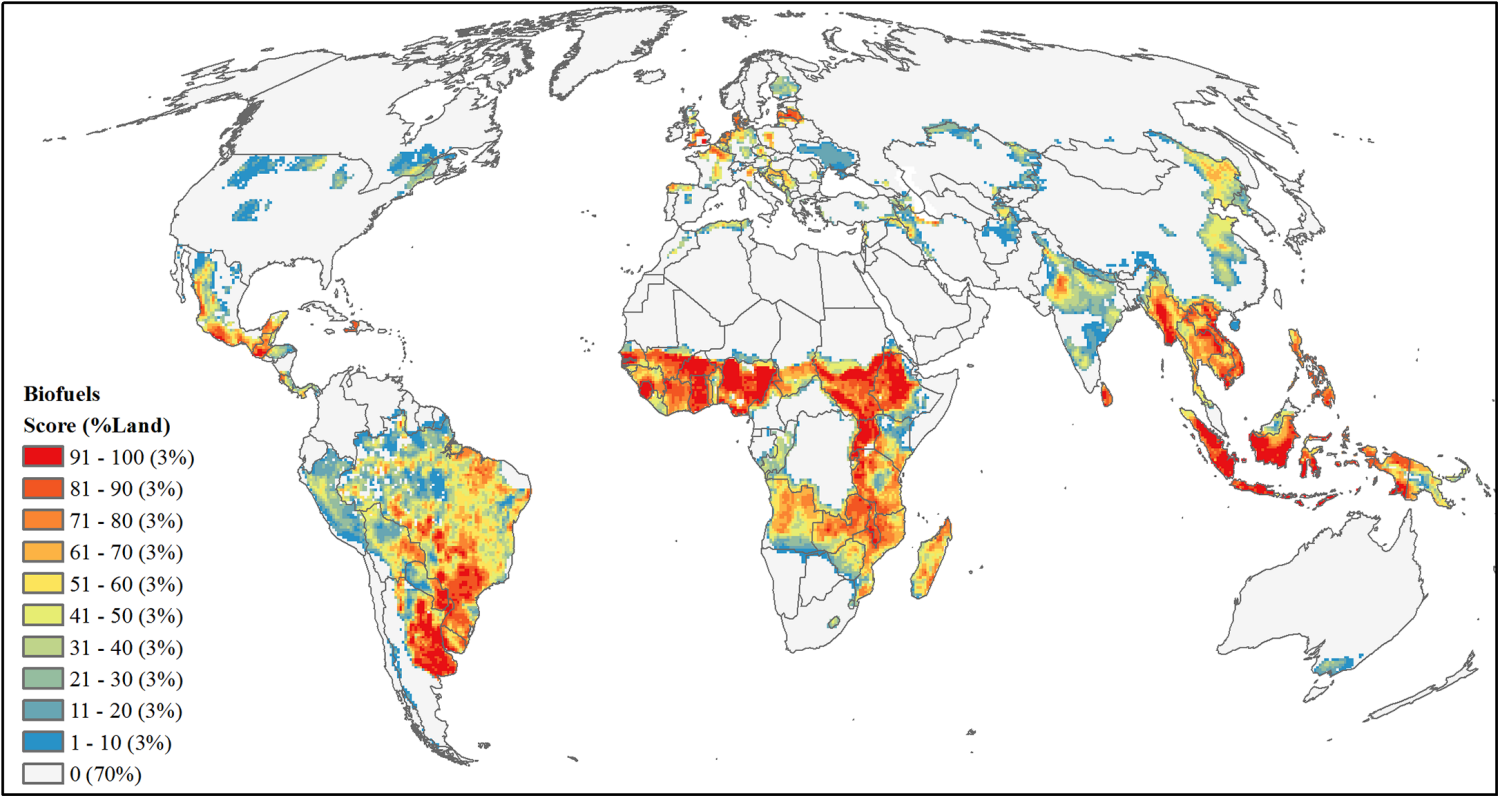
Projected future development threat of first generation biofuels. Area-ranked threat scores based on values of maximum potential gallons of gasoline equivalent multiplied by fraction of agriculture expansion by 2030.

Using crop-specific data for yield and harvested area [79] we focused biofuel production analysis on six first-generation biofuel crops (maize, soybean, sugarcane, rapeseed, sunflower, and oil palm), which make up the vast majority of commercial biofuel production [92] and have mature commercial markets, well-understood technologies, and therefore the potential to accelerate indirect land use change [93]. We assessed development threat by combining the maps of fractional area of cropland expansion by 2030 as described above (see *Agriculture expansion*) with maps of potential biofuel production measured in gallons of gasoline equivalents (GGE). To derive the latter, we first defined 100 crop-specific climate bins based on temperature and precipitation. To capture a range of yields within each climate bin, each climate bin had 1% of the total harvested area for each crop. Within each bin, the maximum potential yield (tons/ha) was defined as the area-weighted 95^th^ percentile yield (i.e. 95% of harvested area within that bin had a lower yield). This methodology is described in more detail in Licker et al. [94] and Mueller et al. [95]. Yields were converted to GGEs using defined values (S3 Table). We then estimated potential expansion of each biofuel crop by mapping the full extent of the 100 crop-specific climate bins in the previous step. The driest climate bin at each temperature range was removed from the analysis as these bins represent extreme growing conditions requiring very intensive irrigation (e.g., Sahara Desert, or interior Australia) and are less likely to be developed. We then generated a maximum potential GGE map (10-km resolution) by combining all six biofuel crops while maintaining the highest GGE value for grid cells where crops overlapped. For final biofuel development threat, we multiplied the potential GGE map by the fractional area of cropland expansion by 2030, resampled the result to a 50-km resolution grid summing potential GGE values, and area-ranked the summed values (Fig 13).

#### Mining

**Fig 14:**
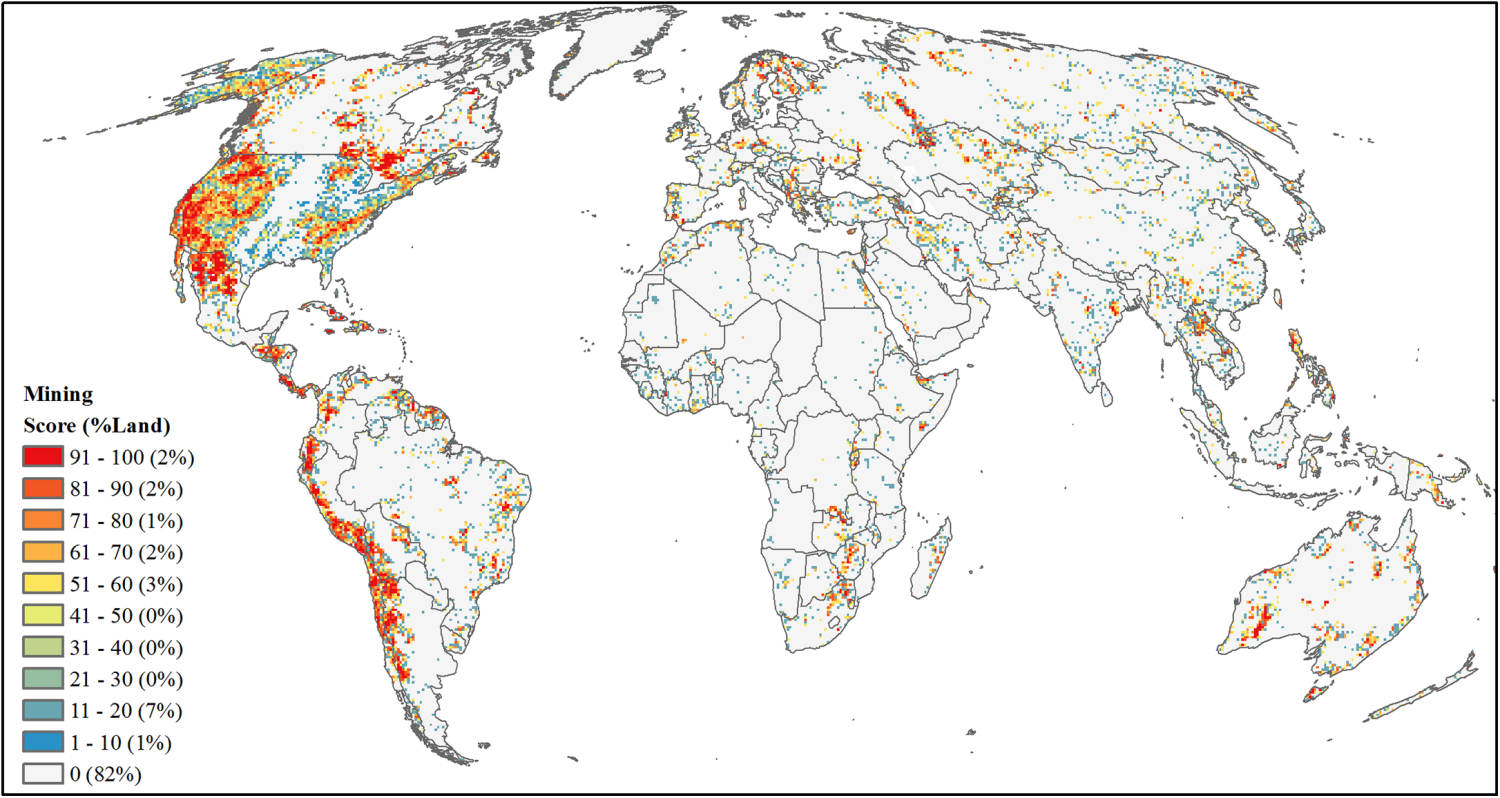
Projected future development threat of mining. Area-ranked threat scores based on number of minerals and geologic materials deposit occurrences and prospects.

We combined three main sources (S1 Table) to identify unexploited mineral deposits and quantify mining development threat: 1) Global Mineral Resources Data System [80], 2) Global Minerals Deposits update of 2011 [81–83] and 3) World Geoscience Database [84]. To discern patterns of future potential development, we removed current or past mining locations and any duplicate locations of the same mineral, resulting in a global dataset of occurrence or prospect deposits (*n* = 116,594). We created a global map that summed the number of unexploited deposits within a 50-km^2^ cell grid. Due to sampling bias towards the U.S. where 74% of deposits occurred, we area-ranked the number of mining occurrences within the U.S. separately from non-U.S. regions and merged the resulting grids into a final mining development threat map (Fig 14).

#### Calculating cumulative and high global development threat

We summed the nine area-ranked sector threat maps (of which each were scaled from 1–100) into one cumulative global threat map. Those cells within our analysis without any threat values were assigned a threat score of zero. We then defined high threat areas as the top summed cells covering 25% of the Earth’s land area (excluding Antarctica).

### Global prioritization of development risk to natural habitats

We defined areas under high development risk as natural habitat which overlapped high cumulative threat from all nine sectors. To classify lands as natural habitat (vs. human-dominated) we used four global datasets; land cover [69], croplands [35], nighttime lights [96], and roads [71]. We reclassified the 300-m resolution land cover data into five classes: water, artificial areas, crops, semi-natural, and natural (S4 Table). We removed all cells classified as water and grouped artificial areas and crops in to one converted class. Using the croplands specific dataset that identifies the proportion of a10-km^2^ cell in agriculture, we further refined our three remaining classes of converted, semi-natural, and natural. We switched any cell classified as natural to a converted class if that cell overlapped a cropland cell having a proportion value > 0.995, and switched those semi-natural cells that overlapped cropland cells having proportion values > 0.5. Conversely, any converted or semi-natural cells that overlapped cropland cells having proportion values < 0.005 and < 0.5, respectively, were considered as natural. Finally with this simplistic land cover dataset (i.e. converted or natural), we considered any cell converted if it overlapped a binary raster (300-m resolution) depicting any lit area [96] or roads [71].

Our analysis identified 76% of the Earth’s land (excluding Antarctica) as natural habitat. This estimate is higher relative to previous ones that range from 50–80% [35,97,98] mainly because we used an additional filtering procedure to include rangeland and semi-natural areas as natural given that they can support diverse, native species [99–101] and partially due to our inclusion of rock and ice areas (e.g. Greenland) often removed when calculating overall percentages [102]. When looking at only ice-free lands our analysis showed 27% being human-dominated which were similar results to Ellis *et al* [98] showing 25% of ice-free land being either densely settled or croplands and nearly matching Hooke *et al* [97] when combining mostly natural lands (46.5%) and mostly uncultivated meadows and pastures (25.8%).

We then selected those 300-m resolution cells identified as natural lands that had cell-centroids falling within our high, cumulative development threat areas (discussed previously), and found that 20% of the global, natural habitat were at risk of future development. To understand if any threats were significant drivers for development risk in any of the geopolitical regions (as defined by ref [16]) or global biomes (as defined by ref [17]) we calculated mean threat scores for each threat restricting this calculation to only natural lands with development risk (S5–S7 Tables).

**Fig 15:**
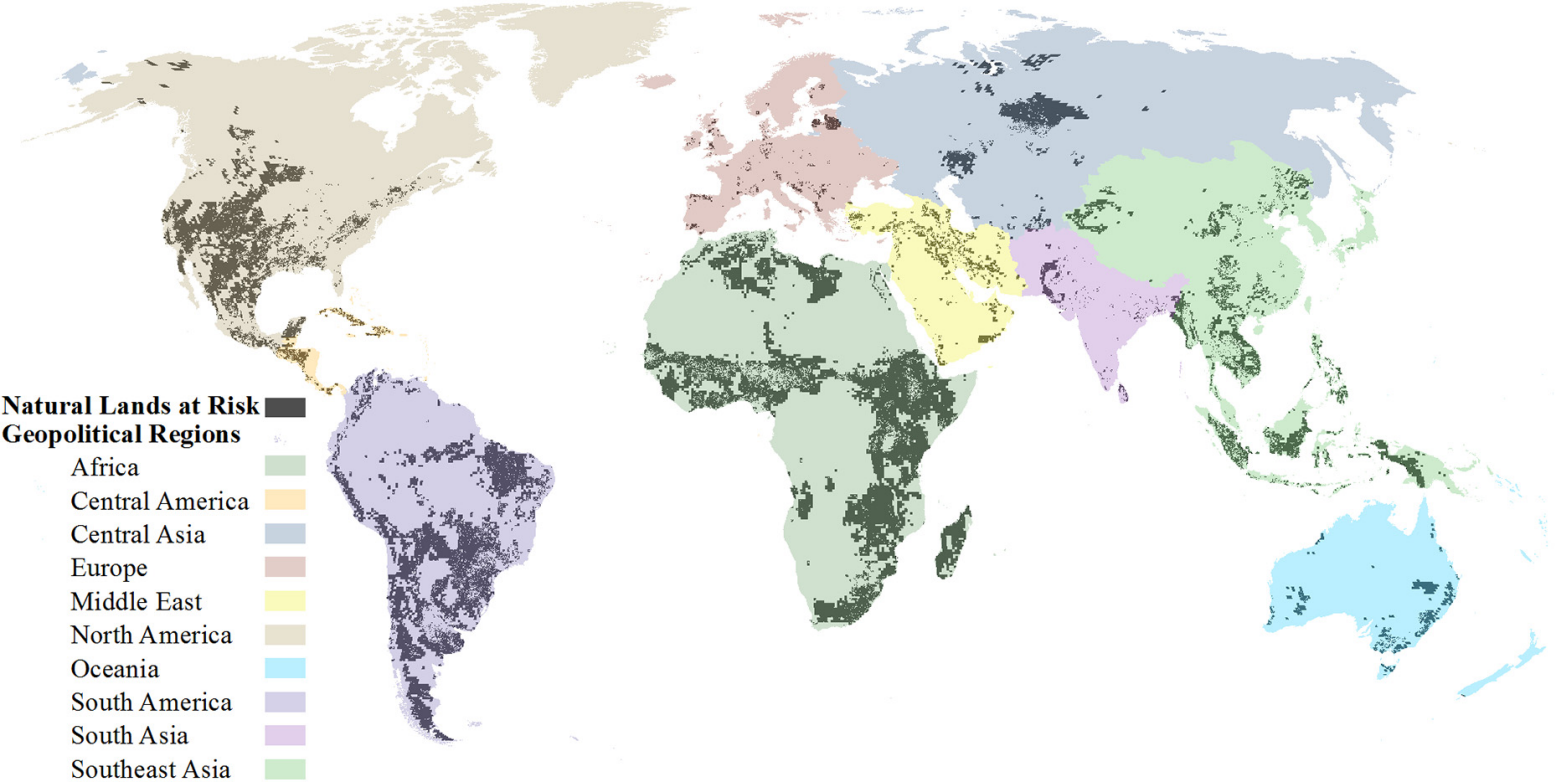
Natural lands at risk within geopolitical regions. Global natural lands at high risk to future development (dark grey) overlaid on geopolitical regions of the world.

**Fig 16:**
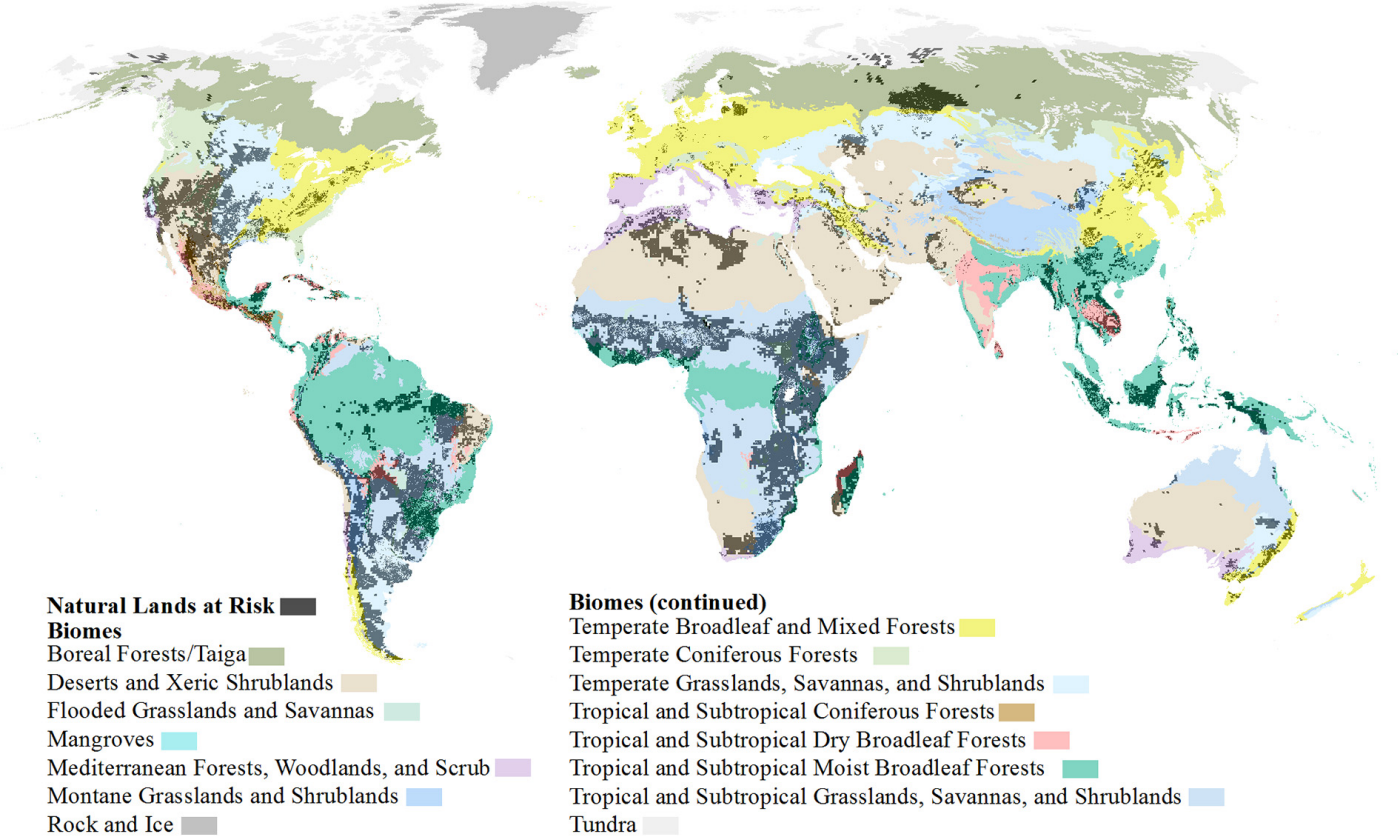
Natural lands at risk within biomes. Global natural lands at high risk to future development (dark grey) overlaid on terrestrial biomes of the world.

For a global prioritization method, we then calculated the square kilometers of land currently converted, currently natural habitat, and at-risk of future development per geopolitical region (Fig 15 and S8 Table) and per biome (Fig 16 and S9 Table). From these values, we were also able to calculate the proportion of each per biome or region and the proportion of at-risk natural lands. To provide a more refined-scaled prioritization, we followed the same procedures and calculated all the above mentioned land amounts and proportions based on ecoregions (as defined by ref [17]). Due to the cumulative development threat analysis extent, 737 ecoregions were examined out of a total of 825 (S1 Dataset). Ecoregions were removed if less than 50% of the ecoregion was covered by the cumulative development threat analysis which eliminated some small island and/or narrow coastal ecoregions (*n* = 86). Additionally the ecoregions, classified as “Rock and Ice” and “Lake”, were removed since our analysis was intended to have a terrestrial focus. To identify potential development restrictions, we also intersected our natural lands at risk to development with strictly protected areas [33], as defined by IUCN category 1–4 [19], and calculated for all three prioritization regions (i.e. geo-political regions, biomes, and ecoregions) the amount and proportion of at-risk lands which are strictly protected.

## Supporting Information

S1 Dataset Development risk for ecoregions. Area and percentages per ecoregion of current land converted, natural lands under high development threat, and strict legal protection of natural lands at-risk.
(XLSX)

S1 Table Source data descriptions and access. Descriptions of data sources used to locate unexploited or potential resources and/or proportions of land predicted to be modified to support future development.
(DOCX)

S2 Table Coal data sources. Data sources used to spatially map coal basins.
(DOCX)

S3 Table GGE values for biofuels. Crop yields (tons) to fuel (gasoline gallon equivalents) conversions applied for biofuel threat.
(DOCX)

S4 Table GlobCov reclassification. Reclassification categories of GlobCov V2 land cover data with original and reclassified values.
(DOCX)

S5 Table Geopolitical region threats per sector. Mean development threat scores per geopolitical region for natural lands at high risk to cumulative development.
(DOCX)

S6 Table Biome threats per sector. Mean development threat scores per biome for natural lands at high risk to cumulative development.
(DOCX)

S7 Table Biome threats ranked by sector. Ranking of development sectors based on mean development threat scores per biome for natural lands at high risk to cumulative development.
(DOCX)

S8 Table Development risk for geopolitical regions. Area and percentages per geopolitical region of current land converted, natural lands under high development threat, and strict legal protection of natural lands at-risk.
(DOCX)

S9 Table Development risk for biomes. Area and percentages per biome of current land converted, natural lands under high development threat, and strict legal protection of natural lands at-risk.
(DOCX)
