# Supplementary material for: A World at Risk: Aggregating Development Trends to Forecast Global Habitat Conversion
Source: PLoS One. 2015 Oct 7;10(10):e0138334. doi: 10.1371/journal.pone.0138334 (PMC4596827; doi:10.1371/journal.pone.0138334)
Supplement: S1 Table — Descriptions of data sources used to locate unexploited or potential resources and/or proportions of land predicted to be modified to support future development. (DOCX) [file pone.0138334.s002.docx]

**S1 Table. Source data descriptions and access.** Descriptions of data sources used to locate unexploited or potential resources and/or proportions of land predicted to be modified to support future development.

| **Threat Category** | **Data Distribution and Access**  *Dataset Name*:   - Data Provider - Dataset Link or Method Accessed - Date Accessed - Citation number associated with bibliography below table | **Data Description**  *Dataset Name*:   - Data Type - Scale or Resolution - Resource values used in analysis - Data reference year |
| --- | --- | --- |
| Urban expansion | *Urban expansion forecast to 2030*:   - Yale University’s Seto Lab - <http://urban.yale.edu/data> - Accessed: 02/12/2014 - Citation: [1] | *Urban expansion forecast to 2030*:   - Type: Raster - Resolution: 5 km - Value: Probability of expansion - Reference Date: 2012 |
| Agriculture expansion | *Historic Cropland and Pasture 2000 -2011:*   - McGill University’s Land Use and the Global Environment Lab - Personal Contact - Accessed: 04/15/2014 - Citation: Unpublished update to [2] | *Historic Cropland and Pasture 2000 - 2011:*   - Type: Raster - Resolution: 5 arc minute (~10km) - Value: Proportion of cell cropland and pasture - Reference Date: 2000 - 2011 |
| Conventional oil and gas | *2000 World Petroleum Assessment - Geologic Provinces of the World*   - *US Geologic Survey (USGS)* - [*http://certmapper.cr.usgs.gov/data/wep/dds60/wep_prvg.zip*](http://certmapper.cr.usgs.gov/data/wep/dds60/wep_prvg.zip) - *Accessed: 09/21/13* - *Citation: [3]*   *2012 World Petroleum Assessment – Province Summary*   - *US Geologic Survey (USGS)* - [*http://pubs.usgs.gov/dds/dds-069/dds-069-ff/downloads/Excel tables/Province Summary.xlsx*](http://pubs.usgs.gov/dds/dds-069/dds-069-ff/downloads/Excel%20tables/Province%20Summary.xlsx) - *Accessed: 12/12/13* - *Citation:[4]* | *2000 World Petroleum Assessment - Geologic Provinces of the World*   - Type: Vector Polygons - Scale: 1: 5,000,000 - Values: Province name, province id, total mean undiscovered petroleum resources (million barrels of oil equivalent) - Reference Date: 2000   *2012 World Petroleum Assessment – Province Summary*   - Type: Excel table - Scale: NA - Value: Total BOE Mean (million barrels of oil equivalent) - Reference Date: 2013 |
| Conventional oil and gas (cont.) | *US National Oil and Gas Assessments 2013*   - US Geologic Survey (USGS) - <http://certmapper.cr.usgs.gov/data/noga00/natl/tabular/2013/Summary_13_Final.xls> - Accessed: 9/3/13 - Citation number [5]   *Petroleum Reserves by Basin as at 1 Jan 2011- Category 2*   - Geoscience Australia - <http://www.ga.gov.au/data-pubs/data-and-publications-search/publications/oil-gas-resources-australia/2010/reserves/table-1> - Accessed: 01/15/2014 - Citation: [6] | *US National Oil and Gas Assessments 2013*   - Type: Excel table - Scale: NA - Values: Conventional Oil mean (billions of barrels), Conventional Gas mean (trillions of cubic feet), Natural Gas Liquids mean (billions of barrels) - Reference Date: 2013   *Petroleum Reserves by Basin as at 1 Jan 2011- Category 2*   - Type: Excel table - Scale: NA - Values: Crude oil (millions of barrels), Sales gas (trillions of cubic feet), LPG (millions of barrels) - Reference Date: 2011 |
| Unconventional oil and gas | *World Shale Gas and Shale Oil Resources*   - US Energy Information Administration - <http://www.eia.gov/analysis/studies/worldshalegas/pdf/fullreport.pdf> - Accessed: October 15, 2013 - Citation: [7]   *US National Oil and Gas Assessments 2013*   - US Geologic Survey (USGS) - <http://certmapper.cr.usgs.gov/data/noga00/natl/tabular/2013/Summary_13_Final.xls> - Accessed: 9/3/13 - Citation: [5] | *World Shale Gas and Shale Oil Resources*   - Type: Maps and Tables in PDF - Scale: Variable - Values: Risked Technically Recoverable Resource Oil (millions of barrels) and Natural Gas (trillions of cubic feet) - Reference Date: 2013   *US National Oil and Gas Assessments 2013*   - Type: Excel table - Scale: NA - Values: Continuous Shale Gas (trillions of cubic feet) and Continuous Oil Gas mean (millions of barrels) - Reference Date: 2013 |
| Coal | *Global Coal Basins*   - (see S2 Table ) - All data accessed from 11/1/2013 to 12/20/2013   *Global Coal Reserves*   - US Energy Information Administration - <http://www.eia.gov/cfapps/ipdbproject/IEDIndex3.cfm?tid=1&pid=7&aid=6> - Accessed: 01/20/14 - Citation: [8]   *US Coal Reserves*   - US Energy Information Administration - <http://www.eia.gov/totalenergy/data/annual/showtext.cfm?t=ptb0408> - Accessed: 01/20/14 - Citation: [9]   *India Coal Reserves*   - Wuppertal Institute - <http://epub.wupperinst.org/frontdoor/index/index/docId/4582> - Accessed: 02/13/14 - Citation: [10]   *China Coal Reserves*   - Wuppertal Institute - <http://epub.wupperinst.org/frontdoor/index/index/docId/4583> - Accessed: 02/13/14 - Citation: [11]   *Australia Coal Reserves*   - Geoscience Australia - <http://epub.wupperinst.org/frontdoor/index/index/docId/4583> - Accessed: 02/20/14 - Citation: [12] | *Global Coal Basins*   - Type: Vector Polygons and digital maps - Scale: Variable - Values: basin area - Date: Varying   *Global Coal Reserves*   - Type: Excel data - Scale: na - Values: coal reserves (short tons) - Reference Date: 2008   *US Coal Reserves*   - Type: Excel table - Scale: na - Values: coal reserves (short tons) - Reference Date: 2011   *India Coal Reserves*   - Type: Table 10-6 in PDF - Scale: na - Values: coal reserves (million tons) - Reference Date: 2010   *China Coal Reserves*   - Type: Table 20-7 in PDF - Scale: na - Values: coal reserves (million tons) - Reference Date: 2006   *Australia Coal Reserves*   - Type: Table 3 in PDF - Scale: na - Values: demonstrated economic reserves (million tons) - Reference Date: 2011 |
| Wind | *Global Wind speed at 80m height*   - 3Tier - <http://maps.google.com/gallery/details?id=zJuaSgXp_WLc.kTBytKPmNODY&hl=en>; geotiff available upon request - Accessed: 03/05/14 - Citation: [13] | *Global Wind speed at 80m height*   - Type: Raster - Resolution: 5km - Values: average annual wind speed (m/s) - Reference Date: based on hourly data 2002 - 2012 |
| Solar | *Global Solar Irradiance*   - 3Tier - <http://maps.google.com/gallery/details?id=zJuaSgXp_WLc.kzhA8d4O7_mk&hl=en>; geotiff available upon request - Accessed: 03/05/14 - Citation: [14] | *Global Solar Irradiance*   - Type: Raster - Resolution: 3km - Values: average annual global horizontal irradiance (W/m^2^) - Reference Date: based on hourly data 2002 - 2012 |
| Biofuels | *Harvested Area and Yields of Six First-Generation Biofuel Crops*   - McGill University’s Land Use and the Global Environment Lab - <http://www.geog.mcgill.ca/landuse/pub/Data/175crops2000/> - Accessed: 04/15/2014 - Citation: [15]   *Historic Cropland 2000 -2011:*   - McGill University’s Land Use and the Global Environment Lab - Personal Contact - Accessed: 04/15/2014 - Citation: Unpublished update to [2] | *Harvested Area and Yields of Six First-Generation Biofuel Crops*   - Type: Raster - Resolution: 5 arc minute (~10km) - Value: Proportion of cell and yield (tons/ha) of specific crop - Reference Date: 2000   *Historic Cropland 2000 - 2011:*   - Type: Raster - Resolution: 5 arc minute (~10km) - Value: Proportion of cell cropland - Reference Date: 2000 -2011 |
| Mining | *Mineral Resources Data System (MRDS)*   - US Geologic Survey (USGS) - <http://mrdata.usgs.gov/mrds/> - Accessed: 06/04/2013 - Citation: [16]   *Global Minerals Deposits Update*   - US Geologic Survey (USGS) - <http://pubs.usgs.gov/of/2008/1155/data/> , <http://pubs.usgs.gov/of/2003/of03-107/data_v1.3/> ,<http://pubs.usgs.gov/of/2009/1045/GIS/> - Accessed: 06/05/2013 - Citations: [17–19]   *World Geoscience Database*   - Geologic Survey of Canada - <http://www.arcgis.com/home/item.html?id=3028f283dab14dd09a7a61b7c9ba0a87> - Accessed: 06/01/2013 - Citation: [20] | *Mineral Resources Data System (MRDS)*   - Type: Vector points - Scale: Variable - Values: Development status - Reference Date: Variable   *Global Minerals Deposits Update*   - Type: Vector points - Scale: Variable - Values: Development status - Reference Date: Variable   *World Geoscience Database*   - Type: Vector points - Scale: Variable - Values: Development status - Reference Date: Variable |

Citation References

1. Seto KC, Güneralp B, Hutyra LR. Global forecasts of urban expansion to 2030 and direct impacts on biodiversity and carbon pools. Proc Natl Acad Sci U S A. 2012;109: 16083–8. doi:10.1073/pnas.1211658109

2. Ramankutty N, Foley JA. Estimating historical changes in global land cover: Croplands from 1700 to 1992. Global Biogeochem Cycles. 1999;13: 997–1027. doi:10.1029/1999GB900046

3. US Geological Survey. World Petroleum Assessment 2000 [Internet]. 2000 [cited 15 Sep 2013]. Available: http://pubs.usgs.gov/dds/dds-060/

4. US Geological Survey. USGS 2012 World Assessment of Undiscovered Oil and Gas Resources [Internet]. 2012 [cited 15 Dec 2013]. Available: http://pubs.usgs.gov/dds/dds-069/dds-069-ff/

5. US Geological Survey. National Oil and Gas Assessment 2012 Assessment Updates [Internet]. 2012 [cited 1 Sep 2013]. Available: http://energy.usgs.gov/OilGas/AssessmentsData/NationalOilGasAssessment/AssessmentUpdates.aspx

6. Geoscience Australia. Petroleum Reserves by Basin as at 1 Jan 2011 [Internet]. 2011 [cited 15 Jan 2014]. Available: http://www.ga.gov.au/products-services/publications/oil-gas-resources-australia/2010/reserves/table-1.html

7. US Energy Information Administration. Technically Recoverable Shale Oil and Shale Gas Resources: An Assessment of 137 Shale Formations in 41 Countries Outside the United States . [Internet]. 2013 [cited 15 Oct 2013]. Available: http://www.eia.gov/analysis/studies/worldshalegas/pdf/fullreport.pdf

8. US Energy Information Administration. Coal Reserves [Internet]. 2008 [cited 15 Jan 2014]. Available: http://www.eia.gov/cfapps/ipdbproject/IEDIndex3.cfm?tid=1&pid=7&aid=6

9. US Energy Information Administration. US Coal Demonstrated Reserve Base, January 1, 2011 (Billion Short Tons) by State [Internet]. 2011 [cited 15 Jan 2014]. Available: http://www.eia.gov/totalenergy/data/annual/showtext.cfm?t=ptb0408

10. Esken A, Höller S, Vallentin D, Viebahn P. CCS global : prospects of carbon capture and storage technologies (CCS) in emerging economies ; final report. Part II: Country study India. 2012.

11. Esken A, Höller S, Vallentin D, Viebahn P. CCS global : prospects of carbon capture and storage technologies (CCS) in emerging economies ; final report. Part III: Country study China. 2012.

12. Geoscience Australia. Australia’s Identified Mineral Resources 2012. 2012.

13. 3TIER by Vaisal. Onshore Global Wind Speeds at 80m Height. 2014.

14. 3TIER by Vaisal. Global Solar Dataset 3km with units in W/m2. 2014.

15. Monfreda C, Ramankutty N, Foley J. Farming the planet: 2. Geographic distribution of crop areas, yields, physiological types, and net primary production in the year 2000. Global Biogeochem Cycles. 2008;22: GB1022–GB1022.

16. US Geological Survey. Mineral Resources Data System (MRDS) [Internet]. 2005 [cited 4 Jun 2013]. Available: http://mrdata.usgs.gov/mrds/

17. Causey JD, Galloway JP, Zientek ML. An index to PGE-Ni-Cr deposits and occurrences in selected mineral-occurrence databases: U.S. Geological Survey Open-File Report 2009-1045 [Internet]. 2009 [cited 15 Jun 2013] p. 19. Available: http://pubs.usgs.gov/of/2009/1045/

18. Cox DP, Lindsey DA, Singer DA, Moring BC, Diggles MF. Sediment-Hosted Copper Deposits of the World: Deposit Models and Database: U.S. Geological Survey Open-File Report 03-107 [Internet]. 2007 [cited 14 Jun 2013] p. 53. Available: http://pubs.usgs.gov/of/2003/of03-107/

19. Singer DA, Berver VI, Moring B. Porphyry copper deposits of the world– Database and grade and tonnage models: U.S. Geological Survey Open-File Report 2008-1155 [Internet]. 2008 [cited 14 Jun 2013] p. 45. Available: http://pubs.usgs.gov/of/2008/1155/

20. Geologic Survey of Canada. World Geoscience Database Projects [Internet]. 2005 [cited 15 Jan 2013]. Available: at http://apps1.gdr.nrcan.gc.ca/gsc_minerals/index.phtml?language=en-CA
