# Supplementary material for: A World at Risk: Aggregating Development Trends to Forecast Global Habitat Conversion
Source: PLoS One. 2015 Oct 7;10(10):e0138334. doi: 10.1371/journal.pone.0138334 (PMC4596827; doi:10.1371/journal.pone.0138334)
Supplement: S2 Table — Data sources used to spatially map coal basins. (DOCX) [file pone.0138334.s003.docx]

**S2 Table. Coal data sources.** Data sources used to spatially map coal basins.

| Country | Spatial Data Source (number associated with bibliography below table) | Data Type |
| --- | --- | --- |
| United States | [1] | Spatial data |
| Russia | [2] | Spatial data |
| China | [3] | Spatial data |
| Australia | [4] | Spatial data |
| India | [5] | Spatial data |
| Germany | [6] | Spatial data |
| Ukraine | [2] | Spatial data |
| Kazakhstan | [2] | Spatial data |
| South Africa | [7] | Spatial data |
| Serbia | [6] | Digital Map |
| Colombia | [1] | Spatial data |
| Canada | [1] | Spatial data |
| Poland | [6] | Digital Map |
| Indonesia | [8] | Digital Map |
| Brazil | [1] | Spatial data |
| Greece | [6] | Digital Map |
| Bosnia and Herzegovina | unavailable | NA |
| Mongolia | [9] | Digital Map |
| Bulgaria | [6] | Digital Map |
| Turkey | [6] | Digital Map |
| Pakistan | [10] | Digital Map |
| Uzbekistan | [2] | Spatial data |
| Hungary | [6] | Digital Map |
| Thailand | [11] | Spatial data |
| Mexico | [1] | Spatial data |
| Iran | [12] | Digital Map |
| Czech Republic | [6] | Digital Map |
| Kyrgyzstan | [2] | Spatial data |
| Albania | [13] | Digital Map |
| North Korea | [14] | Digital Map |
| New Zealand | [15] | Digital Map |
| Spain | [6] | Digital Map |
| Laos | unavailable | NA |
| Zimbabwe | [1] | Spatial data |
| Argentina | [1] | Spatial data |
| Venezuela | [1] | Spatial data |
| Tajikistan | [2] | Spatial data |
| Japan | [9] | Digital Map |
| Macedonia | unavailable | NA |
| Philippines | unavailable | NA |
| Bangladesh | [5] | Digital Map |
| Romania | [6] | Digital Map |
| Slovakia | [6] | Digital Map |
| United Kingdom | [6] | Digital Map |
| Slovenia | [6] | Digital Map |
| Mozambique | [7] | Spatial data |
| Georgia | [9] | Digital Map |
| Tanzania | [1] | Spatial data |
| Nigeria | [1] | Spatial data |
| Greenland | [12] | Digital Map |
| Armenia | unavailable | NA |
| Chile | [1] | Spatial data |
| Vietnam | [9] | Digital Map |
| Swaziland | [1] | Spatial data |
| Montenegro | unavailable | NA |
| South Korea | [16] | Digital Map |
| Belarus | [2] | Spatial data |
| Congo (Kinshasa) | [1] | Spatial data |
| Niger | [1] | Spatial data |
| Afghanistan | [17] | Digital Map |
| Algeria | [1] | Spatial data |
| Peru | [1] | Spatial data |
| Botswana | [1] | Spatial data |
| Portugal | unavailable | NA |
| Ecuador | [1] | Spatial data |
| Egypt | [1] | Spatial data |
| Ireland | unavailable | NA |
| Italy | unavailable | NA |
| Zambia | [1] | Spatial data |
| Norway | unavailable | Digital Map |
| Malaysia | unavailable | NA |
| Central African Republic | [1] | Spatial data |
| Burma (Myanmar) | unavailable | NA |
| Malawi | [1] | Spatial data |
| New Caledonia | unavailable | NA |
| Bolivia | [1] | Spatial data |
| Nepal | unavailable | NA |
| Taiwan | [3] | Spatial data |
| Morocco | [7] | Spatial data |

References Cited

1. Tewalt SJ, Kinney SA, Merrill MD. GIS representation of coal-bearing areas in North, Central, and South America: U.S. Geological Survey Open-File Report 2008-1257. 2008.

2. Brownfield M, Steinshouer D, Povarennykh M, Eriomin I, Shpirt M, Meitov Y, et al. Coal Quality and Resources of the Former Soviet Union: U.S. Geological Survey Open-File Report 02-104. 2001.

3. Karlsen A, Schultz A, Warwick P, Podwysocki S, Lovern V. Coal Geology, Landuse, and Human Health in the People’s Republic of China: U.S. Geological Survey Open-File Report 01-318. 2001.

4. Geoscience Australia. Australian Coal Resources, December 2009 [Internet]. 2009 [cited 15 Jan 2014]. Available: http://www.ga.gov.au/metadata-gateway/metadata/record/gcat_69736

5. Trippi MH, Tewalt SJ. Geographic information system (GIS) representation of coal-bearing areas in India and Bangladesh: U.S. Geological Survey Open-File Report 2011–1296. 2011.

6. EURACOAL - European Association for Coal and Lignite. Country Profiles [Internet]. [cited 1 Apr 2014]. Available: http://www.euracoal.org/pages/layout1sp_graphic.php?idpage=15

7. Merrill MD, Tewalt SJ. GIS representation of coal-bearing areas in Africa: U.S. Geological Survey Open-File Report 2008-1258. 2008.

8. Indonesian COAL.COM. Future Prospects of the Indonesian Coal Mining Sector | Indonesian Coal [Internet]. [cited 1 Apr 2014]. Available: http://indonesiancoal.com/article/future-prospects-of-the-indonesian-coal-mining-sector/

9. Global Methane Initiative. Coal Mine Methane Country Profiles [Internet]. [cited 1 Apr 2014]. Available: https://www.globalmethane.org/

10. Esken A, Höller S, Vallentin D, Viebahn P. CCS global : prospects of carbon capture and storage technologies (CCS) in emerging economies ; final report. Part II: Country study India. 2012.

11. Department of Mineral Resources - Thialand Government. Geology and Mineral Resources of Thailand [Internet]. [cited 1 Apr 2014]. Available: http://www.dmr.go.th/main.php?filename=web_en

12. Landis ER, Weaver JN. Global Coal Occurrence: Chapter 1. 1993.

13. Albania Energy Association. Mineral Resources and Mining Activity in Albania [Internet]. [cited 7 Apr 2014]. Available: http://aea-al.org/mineral-resources-and-mining-activity-in-albania/

14. North Korea Economic Activity [Internet]. [cited 1 Apr 2014]. Available: http://www.lib.utexas.edu/maps/middle_east_and_asia/north_korea_econ_1972.jpg

15. New Zealand - Petroleum and Minerals. New Zealand Coal Fields [Internet]. [cited 1 Apr 2014]. Available: http://www.nzpam.govt.nz/cms/pdf-library/coal-1/map-coaldep.pdf

16. South Korea: Economic Activity [Internet]. [cited 1 Apr 2014]. Available: http://www.lib.utexas.edu/maps/middle_east_and_asia/south_korea_econ_1973.jpg

17. US Geological Survey, US Agency for International Development. Assessing the coal Resources of Afghanistan [Internet]. 2005 [cited 1 Apr 2014]. Available: http://escweb.wr.usgs.gov/share/mooney/USGS coal assessment.pdf
