# Supplementary material for: A World at Risk: Aggregating Development Trends to Forecast Global Habitat Conversion
Source: PLoS One. 2015 Oct 7;10(10):e0138334. doi: 10.1371/journal.pone.0138334 (PMC4596827; doi:10.1371/journal.pone.0138334)
Supplement: S3 Table — Crop yields (tons) to fuel (gasoline gallon equivalents) conversions applied for biofuel threat. (DOCX) [file pone.0138334.s004.docx]

**S3 Table. GGE values for biofuels.** Crop yields (tons) to fuel (gasoline gallon equivalents) conversions applied for biofuel threat.

| **Crop Name** | **Ethanol (liter/ton)*** | **biodiesel (liter/ton)*** | **Conversion (liter to gallon)** | **Biofuels to GGE **** | **Conversion tons to GGE** |
| --- | --- | --- | --- | --- | --- |
| Maize | 410 |  | 0.264172 | 1.5 | 162.47 |
| Sugarcane | 81 |  | 0.264172 | 1.5 | 32.10 |
| Oil Palm |  | 223 | 0.264172 | 0.96 | 56.55 |
| Rapeseed |  | 392 | 0.264172 | 0.96 | 99.41 |
| Soybean |  | 183 | 0.264172 | 0.96 | 46.41 |
| Sunflower |  | 418 | 0.264172 | 0.96 | 106.01 |

* Johnston M, Licker R, Foley JA, Holloway T, Mueller N, Barford C, et al. Closing the gap: global potential for increasing biofuels production through intensification. Environ Res Lett. 2011;6: 34028.

** Fuel Energy Comparisions: Gasoline Gallon Equivalents (GGE) [Internet]. [cited 11 Apr 2014]. Available: http://alternativefuels.about.com/od/resources/a/gge.htm
