# Supplementary material for: A World at Risk: Aggregating Development Trends to Forecast Global Habitat Conversion
Source: PLoS One. 2015 Oct 7;10(10):e0138334. doi: 10.1371/journal.pone.0138334 (PMC4596827; doi:10.1371/journal.pone.0138334)
Supplement: S4 Table — Reclassification categories of GlobCov V2 land cover data with original and reclassified values. (DOCX) [file pone.0138334.s005.docx]

**S4 Table. GlobCov reclassification.** Reclassification categories of GlobCov V2 land cover data with original and reclassified values.

| GlobCov V2 Land Cover Classes* | Reclassified Category |
| --- | --- |
| Post-flooding or irrigated croplands | Crops |
| Rainfed croplands | Crops |
| Mosaic Cropland (50-70%) / Vegetation (grassland, shrubland, forest) (20-50%) | Crops |
| Mosaic Vegetation (grassland, shrubland, forest) (50-70%) / Cropland (20-50%) | Semi-Natural |
| Closed to open (>15%) broadleaved evergreen and/or semi-deciduous forest (>5m) | Natural |
| Closed (>40%) broadleaved deciduous forest (>5m) | Natural |
| Open (15-40%) broadleaved deciduous forest (>5m) | Natural |
| Closed (>40%) needleleaved evergreen forest (>5m) | Natural |
| Open (15-40%) needleleaved deciduous or evergreen forest (>5m) | Natural |
| Closed to open (>15%) mixed broadleaved and needleleaved forest (>5m) | Natural |
| Mosaic Forest/Shrubland (50-70%) / Grassland (20-50%) | Natural |
| Mosaic Grassland (50-70%) / Forest/Shrubland (20-50%) | Natural |
| Closed to open (>15%) shrubland (<5m) | Natural |
| Closed to open (>15%) grassland | Natural |
| Sparse (>15%) vegetation (woody vegetation, shrubs, grassland) | Natural |
| Closed (>40%) broadleaved forest regularly flooded - Fresh water | Natural |
| Closed (>40%) broadleaved semi-deciduous and/or evergreen forest regularly flooded - Saline water | Natural |
| Closed to open (>15%) vegetation (grassland, shrubland, woody vegetation) on regularly flooded or waterlogged soil - Fresh, brackish or saline water | Natural |
| Artificial surfaces and associated areas (urban areas >50%) | Artificial |
| Bare areas | Natural |
| Water bodies | Water |
| Permanent snow and ice | Natural |

***** European Space Agency. GlobCover Land Cover v2 2008 database [Internet]. 2008 [cited 15 Feb 2012]. Available: http://www.esa.int/spaceinimages/Images/2008/12/Envisat_global_land_cover_map
