# Supplementary material for: A World at Risk: Aggregating Development Trends to Forecast Global Habitat Conversion
Source: PLoS One. 2015 Oct 7;10(10):e0138334. doi: 10.1371/journal.pone.0138334 (PMC4596827; doi:10.1371/journal.pone.0138334)
Supplement: S5 Table — Mean development threat scores per geopolitical region for natural lands at high risk to cumulative development. (DOCX) [file pone.0138334.s006.docx]

**S5 Table. Geopolitical region threats per sector.** Mean development threat scores per geopolitical region for natural lands at high risk to cumulative development.

| Regions | Urban | Ag | Conv. Oil and Gas | Unconv. Oil and Gas | Coal | Wind | Solar | Biofuels | Mining |
| --- | --- | --- | --- | --- | --- | --- | --- | --- | --- |
| Africa | 46.88 | 64.56 | 55.27 | 75.62 | 29.18 | 52.67 | 74.74 | 68.98 | 42.02 |
| Central America | 51.63 | 68.39 | 73.36 | 0.00 | 0.00 | 54.61 | 46.58 | 58.83 | 73.58 |
| Central Asia | 56.40 | 43.63 | 96.40 | 92.32 | 86.95 | 55.82 | 10.29 | 28.11 | 61.85 |
| Europe | 44.55 | 73.73 | 32.21 | 21.26 | 35.54 | 65.23 | 22.22 | 59.94 | 58.94 |
| Middle East | 55.03 | 58.86 | 55.13 | 31.41 | 39.73 | 57.55 | 69.42 | 32.31 | 46.84 |
| North America | 37.58 | 53.54 | 38.17 | 31.93 | 57.49 | 67.11 | 62.44 | 33.77 | 59.58 |
| Oceania | 49.79 | 76.57 | 10.60 | 58.84 | 74.79 | 63.16 | 57.06 | 11.80 | 66.57 |
| South America | 43.47 | 60.19 | 54.97 | 52.29 | 24.98 | 66.66 | 52.88 | 57.36 | 63.84 |
| South Asia | 54.85 | 56.21 | 66.74 | 75.13 | 44.74 | 66.54 | 60.00 | 45.87 | 49.95 |
| Southeast Asia | 44.00 | 67.34 | 68.32 | 59.15 | 51.60 | 59.81 | 28.16 | 76.16 | 47.19 |
