# Supplementary material for: A World at Risk: Aggregating Development Trends to Forecast Global Habitat Conversion
Source: PLoS One. 2015 Oct 7;10(10):e0138334. doi: 10.1371/journal.pone.0138334 (PMC4596827; doi:10.1371/journal.pone.0138334)
Supplement: S6 Table — Mean development threat scores per biome for natural lands at high risk to cumulative development. (DOCX) [file pone.0138334.s007.docx]

**S6 Table. Biome threats per sector.** Mean development threat scores per biome for natural lands at high risk to cumulative development.

| Biome Name | Urban | Ag. | Conv. Oil and Gas | Unconv. Oil and Gas | Coal | Wind | Solar | Biofuels | Mining |
| --- | --- | --- | --- | --- | --- | --- | --- | --- | --- |
| Boreal Forests/Taiga | 51.67 | 41.16 | 97.45 | 93.48 | 92.08 | 52.40 | 0.00 | 33.47 | 60.79 |
| Deserts and Xeric Shrublands | 46.87 | 53.49 | 60.57 | 64.17 | 49.87 | 56.83 | 70.17 | 30.49 | 63.77 |
| Flooded Grasslands and Savannas | 44.33 | 60.74 | 69.68 | 43.04 | 38.00 | 31.21 | 53.04 | 73.80 | 36.25 |
| Mangroves | 60.99 | 72.35 | 73.13 | 37.23 | 36.07 | 35.22 | 43.08 | 74.43 | 45.77 |
| Mediterranean Forests, Woodlands, and Scrub | 51.48 | 56.58 | 32.65 | 57.86 | 50.34 | 68.72 | 69.95 | 26.25 | 64.79 |
| Montane Grasslands and Shrublands | 44.47 | 49.00 | 36.29 | 87.85 | 45.72 | 62.59 | 68.25 | 48.47 | 72.46 |
| Temperate Broadleaf and Mixed Forests | 46.01 | 63.47 | 50.77 | 55.03 | 53.61 | 67.22 | 40.26 | 41.37 | 49.54 |
| Temperate Coniferous Forests | 38.65 | 46.40 | 39.64 | 32.30 | 53.03 | 69.90 | 47.49 | 13.31 | 63.26 |
| Temperate Grasslands, Savannas, and Shrublands | 38.86 | 61.39 | 34.03 | 43.36 | 62.50 | 80.33 | 40.62 | 46.22 | 42.58 |
| Tropical and Subtropical Coniferous Forests | 53.40 | 63.43 | 44.96 | 46.03 | 13.80 | 57.80 | 66.37 | 49.81 | 67.39 |
| Tropical and Subtropical Dry Broadleaf Forests | 43.08 | 62.57 | 68.82 | 55.12 | 21.82 | 53.11 | 61.76 | 66.76 | 53.00 |
| Tropical and Subtropical Grasslands, Savannas, and Shrublands | 42.11 | 65.80 | 52.74 | 46.04 | 23.68 | 49.94 | 71.27 | 68.88 | 44.01 |
| Tropical and Subtropical Moist Broadleaf Forests | 49.75 | 67.50 | 60.62 | 46.94 | 46.08 | 53.36 | 49.23 | 68.71 | 52.20 |
| Tundra | 60.95 | 61.00 | 92.67 | 64.28 | 69.37 | 55.87 | 0.00 | 0.00 | 58.78 |
