# Supplementary material for: A World at Risk: Aggregating Development Trends to Forecast Global Habitat Conversion
Source: PLoS One. 2015 Oct 7;10(10):e0138334. doi: 10.1371/journal.pone.0138334 (PMC4596827; doi:10.1371/journal.pone.0138334)
Supplement: S7 Table — Ranking of development sectors based on mean development threat scores per biome for natural lands at high risk to cumulative development. (DOCX) [file pone.0138334.s008.docx]

**S7 Table. Biome threats ranked by sector.** Ranking of development sectors based on mean development threat scores per biome for natural lands at high risk to cumulative development.

| Biome Name | Urban | Ag. | Conv. Oil and Gas | Unconv. Oil and Gas | Coal | Wind | Solar | Biofuels | Mining |
| --- | --- | --- | --- | --- | --- | --- | --- | --- | --- |
| Boreal Forests/Taiga | 6 | 7 | 1 | 2 | 3 | 5 | 9 | 8 | 4 |
| Deserts and Xeric Shrublands | 8 | 6 | 4 | 2 | 7 | 5 | 1 | 9 | 3 |
| Flooded Grasslands and Savannas | 5 | 3 | 2 | 6 | 7 | 9 | 4 | 1 | 8 |
| Mangroves | 4 | 3 | 2 | 7 | 8 | 9 | 6 | 1 | 5 |
| Mediterranean Forests, Woodlands, and Scrub | 6 | 5 | 8 | 4 | 7 | 2 | 1 | 9 | 3 |
| Montane Grasslands and Shrublands | 8 | 5 | 9 | 1 | 7 | 4 | 3 | 6 | 2 |
| Temperate Broadleaf and Mixed Forests | 7 | 2 | 5 | 3 | 4 | 1 | 9 | 8 | 6 |
| Temperate Coniferous Forests | 7 | 5 | 6 | 8 | 3 | 1 | 4 | 9 | 2 |
| Temperate Grasslands, Savannas, and Shrublands | 8 | 3 | 9 | 5 | 2 | 1 | 7 | 4 | 6 |
| Tropical and Subtropical Coniferous Forests | 5 | 3 | 8 | 7 | 9 | 4 | 2 | 6 | 1 |
| Tropical and Subtropical Dry Broadleaf Forests | 8 | 3 | 1 | 5 | 9 | 6 | 4 | 2 | 7 |
| Tropical and Subtropical Grasslands, Savannas, and Shrublands | 8 | 3 | 4 | 6 | 9 | 5 | 1 | 2 | 7 |
| Tropical and Subtropical Moist Broadleaf Forests | 6 | 2 | 3 | 8 | 9 | 4 | 7 | 1 | 5 |
| Tundra | 5 | 4 | 1 | 3 | 2 | 7 | 8.5 | 8.5 | 6 |
