# Supplementary material for: A World at Risk: Aggregating Development Trends to Forecast Global Habitat Conversion
Source: PLoS One. 2015 Oct 7;10(10):e0138334. doi: 10.1371/journal.pone.0138334 (PMC4596827; doi:10.1371/journal.pone.0138334)
Supplement: S8 Table — Area and percentages per geopolitical region of current land converted, natural lands under high development threat, and strict legal protection of natural lands at-risk. (DOCX) [file pone.0138334.s009.docx]

**S8 Table. Development risk for geopolitical regions.** Area and percentages per geopolitical region of current land converted, natural lands under high development threat, and strict legal protection of natural lands at-risk.

| Region Name | Natural Lands (KM^2^) | Currently Converted Lands (KM^2^) | Percent Currently Converted | Natural Lands at Risk (KM^2^) | Percent Natural Lands at Risk | Percent Converted in Future | Strictly Protected  Natural Lands at Risk (KM^2^) | Percent of Natural Lands at Risk Strictly Protected |
| --- | --- | --- | --- | --- | --- | --- | --- | --- |
| Africa | 26,144,851 | 3,627,614 | 12% | 8,179,901 | 31% | 40% | 502,598 | 6% |
| Central America | 406,222 | 324,628 | 44% | 175,198 | 43% | 68% | 14,811 | 8% |
| Central Asia | 16,762,238 | 3,657,175 | 18% | 868,366 | 5% | 22% | 6,146 | 1% |
| Europe | 1,723,232 | 4,089,560 | 70% | 156,035 | 9% | 73% | 14,125 | 9% |
| Middle East | 4,209,865 | 1,901,877 | 31% | 534,704 | 13% | 40% | 13,990 | 3% |
| North America | 17,625,696 | 4,825,984 | 21% | 3,078,901 | 17% | 35% | 180,176 | 6% |
| Oceania | 7,065,783 | 927,474 | 12% | 410,890 | 6% | 17% | 55,302 | 13% |
| South America | 13,814,394 | 3,768,007 | 21% | 4,322,885 | 31% | 46% | 203,570 | 5% |
| South Asia | 1,828,962 | 3,176,641 | 63% | 204,631 | 11% | 68% | 18,108 | 9% |
| Southeast Asia | 10,519,451 | 5,872,336 | 36% | 1,767,437 | 17% | 47% | 108,790 | 6% |
