# Supplementary material for: A World at Risk: Aggregating Development Trends to Forecast Global Habitat Conversion
Source: PLoS One. 2015 Oct 7;10(10):e0138334. doi: 10.1371/journal.pone.0138334 (PMC4596827; doi:10.1371/journal.pone.0138334)
Supplement: S9 Table — Area and percentages per biome of current land converted, natural lands under high development threat, and strict legal protection of natural lands at-risk. (DOCX) [file pone.0138334.s010.docx]

**S9 Table. Development risk for biomes.** Area and percentages per biome of current land converted, natural lands under high development threat, and strict legal protection of natural lands at-risk.

| Biome Name | Natural Lands (KM^2^) | Currently Converted Lands (KM^2^) | Percent Currently Converted | Natural Lands at Risk (KM^2^) | Percent Natural Lands at Risk | Percent Converted in Future | Strictly Protected  Natural Lands at Risk (KM^2^) | Percent of Natural Lands at Risk  Strictly Protected |
| --- | --- | --- | --- | --- | --- | --- | --- | --- |
| Boreal Forests/Taiga | 13,445,009 | 892,294 | 6% | 530,726 | 4% | 10% | 3,045 | 1% |
| Deserts and Xeric Shrublands | 24,028,625 | 3,553,965 | 13% | 3,737,718 | 16% | 26% | 159,092 | 4% |
| Flooded Grasslands and Savannas | 806,639 | 239,550 | 23% | 337,660 | 42% | 55% | 39,826 | 12% |
| Mangroves | 117,909 | 97,253 | 45% | 57,330 | 49% | 72% | 6,508 | 11% |
| Mediterranean Forests, Woodlands, and Scrub | 1,363,063 | 1,634,973 | 55% | 422,831 | 31% | 69% | 23,795 | 6% |
| Montane Grasslands and Shrublands | 4,379,566 | 767,132 | 15% | 1,170,865 | 27% | 38% | 59,048 | 5% |
| Temperate Broadleaf and Mixed Forests | 4,368,120 | 7,872,298 | 64% | 877,997 | 20% | 71% | 65,437 | 8% |
| Temperate Coniferous Forests | 2,963,970 | 998,423 | 25% | 545,968 | 18% | 39% | 67,097 | 12% |
| Temperate Grasslands, Savannas, and Shrublands | 5,421,978 | 4,481,855 | 45% | 1,610,898 | 30% | 62% | 19,464 | 1% |
| Tropical and Subtropical Coniferous Forests | 473,073 | 226,349 | 32% | 244,265 | 52% | 67% | 6,693 | 3% |
| Tropical and Subtropical Dry Broadleaf Forests | 1,094,337 | 1,785,540 | 62% | 608,856 | 56% | 83% | 48,252 | 8% |
| Tropical and Subtropical Grasslands, Savannas, and Shrublands | 16,744,786 | 3,338,073 | 17% | 5,980,457 | 36% | 46% | 421,028 | 7% |
| Tropical and Subtropical Moist Broadleaf Forests | 13,856,312 | 5,205,978 | 27% | 3,400,648 | 25% | 45% | 179,109 | 5% |
| Tundra | 7,361,183 | 117,967 | 2% | 154,004 | 2% | 4% | 18,006 | 12% |
